# Disparities in Digital Health Care Use in 2022

**DOI:** 10.1001/jamanetworkopen.2025.5359

**Published:** 2025-04-17

**Authors:** Michael Wakeman, Dennis W. Buckman, Sherine El-Toukhy

**Affiliations:** 1 Division of Intramural Research, National Institute on Minority Health and Health Disparities, National Institutes of Health, Rockville, Maryland; 2 Information Management Services, Calverton, Maryland

## Abstract

**Importance:**

Digital health care services expanded with the COVID-19 pandemic. Disparities in telehealth, telemedicine, and telemonitoring use remain understudied.

**Objective:**

To examine associations between individual-level characteristics and digital health care use and if these associations differ by county-level social vulnerability.

**Design, Setting, and Participants:**

This cross-sectional study was an online survey that included a nonprobability sample of US adults aged 18 years or older who resided in 871 counties in the least or most vulnerable quartiles of the Minority Health Social Vulnerability Index (MHSVI), an indicator of county-level social vulnerability. The study was conducted between February and August 2022, and data were analyzed from August 2023 to August 2024.

**Exposures:**

Participant characteristics and MHSVI county-level social vulnerability.

**Main Outcomes and Measures:**

Self-reported use of telehealth, telemedicine, and telemonitoring. Multivariable logistic regression models were fit to examine associations between sociodemographic, health, and technology factors and each service use, overall and stratified by MHSVI.

**Results:**

Of the 5444 participants who were included in this study, 2927 were female (53.77%), 798 were non-Hispanic Black or African American (14.66%), 838 were Hispanic or Latino (15.39%), 3542 were non-Hispanic White (65.06%); the mean (SE) age was 45.4 (0.2) years. Overall, 2754 participants used telehealth (50.59%), 1609 used telemedicine (29.56%), and 854 used telemonitoring (15.69%). Being English nonproficient (adjusted odds ratio [aOR], 1.54; 95% CI, 1.23-1.92) and having had in-person health care visits (aOR, 4.71; 95% CI, 3.93-5.63) were associated with higher odds of using telehealth, whereas not having a primary care clinician was associated with lower odds (aOR, 0.68; 95% CI, 0.59-0.78). Similar findings were documented for telemedicine and telemonitoring use. Education was associated with higher odds of digital health care use in MHSVI most vulnerable counties (telehealth: aOR, 1.18; 95% CI, 1.06-1.32; telemedicine: aOR, 1.18; 95% CI, 1.05-1.33), whereas individuals who did not self-identify as heterosexual (telehealth: aOR, 1.47; 95% CI, 1.10-1.97; telemedicine: aOR, 1.57; 95% CI, 1.16-2.11; telemonitoring: aOR, 1.54; 95% CI, 1.02-2.31) and those who self-reported fair or poor mental health (telehealth: aOR, 1.29; 95% CI, 1.03-1.61) had higher odds of digital service use in the least vulnerable counties. Self-identifying as Black or African American or Hispanic was associated with high odds of telehealth (Black or African American: aOR, 1.41; 95% CI, 1.17-1.70; Hispanic or Latino: aOR, 1.41; 95% CI, 1.17-1.70), telemedicine (Black or African American: aOR, 1.44; 95% CI, 1.18-1.76; Hispanic or Latino: aOR, 1.27; 95% CI, 1.04-1.54), and telemonitoring (Black or African American: aOR, 1.40; 95% CI, 1.11-1.78; Hispanic or Latino: aOR, 1.46; 95%CI, 1.16-1.84) use overall, but these associations varied across MHSVI strata.

**Conclusions and Relevance:**

In this cross-sectional study of US adults from MHSVI most and least vulnerable counties, digital health care use varied by participant characteristics. Some traditionally underserved groups self-reported higher use of digital health care. Differing associations between individual-level characteristics and digital health care use by county-level social vulnerability reflect the importance of place-based disadvantage indicators. Eliminating digital health care use disparities is important as it represents a complementary avenue to access health care for underserved populations.

## Key Points

**Question:**

What individual-level characteristics are associated with telehealth, telemedicine, and telemonitoring use, and do these associations differ by county-level social vulnerability?

**Findings:**

This cross-sectional study of 5444 US adults from Minority Health Social Vulnerability Index most and least vulnerable counties showed that 50.59% used telehealth, 29.56% used telemedicine, and 15.69% used telemonitoring in 2022. Differences in utilization were observed across race and ethnicity, English proficiency, and those seeking in-person care, with some associations varying between most and least vulnerable counties.

**Meaning:**

These findings suggest that digital health care utilization varied by individual- and county-based indicators of disadvantage, although traditionally underresourced groups self-reported higher digital health care use in some cases.

## Teaser

This cross-sectional study examines associations between individuals-level characteristics and digital health care use and evaluates whether these associations differ by county-level social vulnerability using the Minority Health Social Vulnerability Index.

## Introduction

Digital technologies allow for the provision of health care, consultations and clinical services, and continuous monitoring of patients outside traditional clinical settings. Digital health care services, such as telehealth, telemedicine, and telemonitoring, were underused prior to the COVID-19 pandemic.^1^ The adoption of digital health care services increased during the pandemic to curb disease transmission and strain on health care systems while maintaining access to critical services,^2^ facilitated by temporary policies.^3,4^ For example, 95.4% of health centers funded by the Health Resources and Services Administration provided telehealth services in 2020.^5^ While many policies have since expired,^3,6^ digital health care services persisted. For example, telehealth visits of Medicare outpatient services were 3.50% in 2021, 43 times higher than the less than 1% prepandemic levels in 2019.^7^

Digital health care curtails traditional barriers to care.^8^ It is associated with improved patient health outcomes and enhanced management of chronic health conditions. Additionally, digital health care saved time, had more efficient workflows, and provided financial savings for health care systems and patients.^9,10,11,12^ Physicians and patients, including underserved populations, report high rates of satisfaction and willingness to use digital health care.^13,14,15,16^

Disparities in accessing and using digital health care impede the realization of associated benefits. Factors associated with limited digital health care access and use include demographics (eg, being male),^17,18^ digital literacy as evidenced by the modality of services used (ie, audio-only),^18,19,20^ and community-level characteristics (eg, rural vs urban).^21,22,23^

Gaps remain in our understanding of factors associated with telehealth, telemedicine, and telemonitoring use as distinct services and the interplay between individual- and community-level indicators of disadvantage on digital health care services use. This study aimed to identify sociodemographic, health, and technology factors associated with telehealth, telemedicine, and telemonitoring use and assess if these associations varied by county-level social vulnerability.

## Methods

### Study Sample

This cross-sectional study followed the Strengthening the Reporting of Observational Studies in Epidemiology (STROBE) reporting guideline.^24^ This study was deemed exempt from review by the institutional review board at the National Institutes of Health. All participants provided consent to participate in the study at the start of the online survey.

We recruited a nonprobability sample of 5559 US adults, age 18 years or older, residing in the top and bottom quartile counties of the 2018 Minority Health Social Vulnerability Index (MHSVI), to participate in an online survey using Qualtrics’ participant panels. MHSVI is an index of community vulnerability that is based on 34 indicators across 6 themes: socioeconomic status, household composition and disability, minority status and language, housing type and transportation, health care infrastructure and access, and medical vulnerability.^25^ The 2018 MHSVI indicators were based on the 2014 to 2018 estimates of the American Community Survey. To have each theme equally contribute to the overall MHSVI score, we summed the scaled county rankings of each subtheme, then ranked and scaled the final sum between 0 and 1, with higher scores reflecting greater vulnerability. We identified 1571 counties that fell in the top (ie, most vulnerable; n = 786) or bottom (ie, least vulnerable; n = 785) MHSVI quartile. The survey had a 17% participation rate.

Using the National Center for Health Statistics (NCHS) Vintage Bridged-Race Postcensal Population Estimates,^26^ we calculated the sex, age, and racial and ethnic composition of residents in MHSVI top and bottom quartile counties. The sample largely reflected the demographic breakdown of residents in MHSVI strata counties per NCHS’s percentages (eTable 1 in Supplement 1). While results based on nonprobability sampling are nongeneralizable, we recruited as many participants as possible to allow for stratified analyses by MHSVI, inclusion of several factors in planned models, and increased precision for adjusted odds ratio (aOR) and prevalence estimates.

Participants who failed a quality control test (ie, inconsistently reported their age) were excluded (n = 115) (eTable 2 in Supplement 1). The resulting analytic sample included 5444 participants who resided in 871 counties. Data were collected between February 23, 2022, and August 26, 2022.

### Measures

For telehealth, participants were asked “In the past 12 months, not counting times you went to an emergency room, how many times did you have an appointment with a healthcare [clinician] by video or phone?”; for telemedicine, “How many times did you receive remote counseling or remotely supervised training or therapy from a healthcare [clinician] online or by phone?”; and for telemonitoring, “In the past 12 months, did you receive a device (eg, home spirometer, thermometer, fingertip oxygen monitor called oximeter) from your healthcare [clinician] that would allow the medical team to monitor you remotely?”^27,28,29,30^ Responses were dichotomized into 0 times or 1 or more times for telehealth and telemedicine and yes or no for telemonitoring.

We collected data on participants’ age, biological sex, self-identified race and ethnicity, education, income, sexual orientation, English proficiency, general and mental health, health insurance, having a primary care clinician (PCC), underlying medical conditions, past-year in-person visits with health care clinician, and ever testing positive for COVID-19.^30^ Participants self-identified their race and ethnicity from prespecified categories based on standards of the Office of Management and Budget. Sexual orientation choices included heterosexual or straight; homosexual, gay, or lesbian; bisexual; none of these describe me; I do not know; and prefer not to answer. We collected data on risk factors, including body mass index (BMI), physical activity, cigarette smoking, e-cigarette use, alcohol misuse, and marijuana use. All risk factors were dichotomized per health guidelines. Finally, we collected data on internet access and digital health literacy using the eHealth Literacy Questionnaire.^31^

### Statistical Methods

We used fully conditional specification methods implemented in the SAS MI procedure, and under the missing at random assumption, fit imputation models fully adjusted for all study covariates and outcome variables and imputed 25 datasets with imputed data for 6 variables with “do not know,” “prefer not to answer,” or “unknown” responses.^32^ Missingness ranged from 0.79% for education to 13.15% for weight (eTable 2 in Supplement 1). We used multiple imputation methods implemented in SUDAAN to fit multivariable logistic regression models to examine differences in telehealth, telemedicine, and telemonitoring use by sociodemographic, health, and technology factors overall and stratified by MHSVI. Using the fitted models and predictive margins methods implemented in SUDAAN, we generated predictive marginals of the probability of telehealth, telemedicine, and telemonitoring use adjusting for sociodemographic, health, and technology factors.^33^ For the logistic regression models, we report the aOR and predictive marginal probability estimates along with confidence limits. Age and digital health literacy were treated as continuous variables, education and income as ordinal variables, and all others as categorical variables. Non-Hispanic Asian, Native Hawaiian, Pacific Islander, American Indian, and Alaska Native individuals were collapsed into non-Hispanic other. For our analysis, individuals who selected homosexual, gay, lesbian, or bisexual were collapsed into the category not heterosexual. As a sensitivity analysis, we fit additional models using complete case data under the missing completely at random assumption and compared the results with the models fit using multiple imputation data under missing at random assumption. We conducted a post hoc analysis examining factors associated with mutually exclusive use of digital health care services (eTable 3 in Supplement 1). Statistical significance was set at *P* < .05, and statistically significant odds ratios did not include the null value of 1 in the confidence interval. Analyses were conducted using SAS version 9.4 (SAS Institute) and SUDAAN version 11.0.4 (RTI International).^34,35^

## Results

Of 5444 participants, 2927 were female (53.77%), 798 were non-Hispanic Black or African American (14.66%), 838 were Hispanic or Latino (15.39%), and 3542 were non-Hispanic White (65.06%); the mean (SE) age, 45.4 (0.2) years, and 1743 had a high school education or less (32.01%) (Table 1). In total, 2754 participants used telehealth (50.59%), 1609 used telemedicine (29.56%), and 854 used telemonitoring (15.69%).

**Table 1.** Sample Characteristics Stratified by MHSVI Most Vulnerable and Least Vulnerable Counties (N = 5444 Participants, Imputed Data)

Characteristics	Participants, No. (%)			*P* value
	Overall (n = 5444)	MHSVI most vulnerable counties (n = 2794)^a^	MHSVI least vulnerable counties (n = 2650)^a^	
Age, mean (SE), y	45.41 (0.23)	43.46 (0.31)	47.46 (0.34)	
18-29	1222 (22.45)	692 (24.77)	530 (20.00)	<.001^b^
30-44	1517 (27.87)	840 (30.06)	677 (25.55)	
45-59	1387 (25.48)	714 (25.55)	673 (25.40)	
≥60	1318 (24.21)	548 (19.61)	770 (29.06)	
Sex				
Female	2927 (53.77)	1597 (57.16)	1330 (50.19)	<.001^b^
Male	2517 (46.23)	1197 (42.84)	1320 (49.81)	
Sexual orientation				
Heterosexual	4833 (88.78)	2464 (88.20)	2369 (89.38)	.17
Not heterosexual^c^	611 (11.22)	330 (11.80)	281 (10.62)	
Race and ethnicity				
Hispanic or Latino	838 (15.39)	643 (23.01)	195 (7.36)	<.001^b^
Non-Hispanic American Indian or Alaska Native	58 (1.07)	51 (1.83)	7 (0.26)	
Non-Hispanic Asian, Native Hawaiian or Pacific Islander	208 (3.82)	75 (2.68)	133 (5.02)	
Non-Hispanic Black or African American	798 (14.66)	634 (22.69)	164 (6.19)	
Non-Hispanic White	3542 (65.06)	1391 (49.79)	2151 (81.17)	
Education				
Less than high school	284 (5.22)	197 (7.04)	87 (3.30)	<.001^b^
High school graduate	1459 (26.80)	868 (31.06)	591 (22.31)	
Vocational school, some college	1992 (36.58)	1076 (38.49)	916 (34.57)	
College graduate or higher	1709 (31.39)	654 (23.41)	1055 (39.82)	
Income (in 2021)				
<$20 000	1330 (24.42)	896 (32.08)	433 (16.35)	<.001^b^
$20 000 to $49 999	1710 (31.41)	993 (35.54)	717 (27.06)	
$50 000 to $74 999	949 (17.43)	414 (14.82)	535 (20.18)	
≥$75 000	1456 (26.74)	491 (17.56)	965 (36.41)	
English proficiency				
Very well	4984 (91.55)	2525 (90.37)	2459 (92.79)	.001^b^
Well, not well, not at all	460 (8.45)	269 (9.63)	191 (7.21)	
Health insurance				
Insured	4844 (88.98)	2371 (84.86)	2473 (93.32)	<.001^b^
Uninsured	600 (11.02)	423 (15.14)	177 (6.68)	
General health				
Excellent, very good, good	4144 (76.12)	2072 (74.16)	2072 (78.19)	<.001^b^
Fair, poor	1300 (23.88)	722 (25.84)	578 (21.81)	
Mental health				
Excellent, very good, good	3972 (72.96)	1982 (70.94)	1990 (75.09)	<.001^b^
Fair, poor	1472 (27.04)	812 (29.06)	660 (24.91)	
Having a primary care clinician				
Yes	2609 (47.92)	1248 (44.67)	1361 (51.36)	<.001^b^
No	2835 (52.08)	1546 (55.33)	1289 (48.64)	
Presence of underlying medical conditions				
Yes	1887 (34.66)	914 (32.71)	973 (36.72)	.001^b^
No	3557 (65.34)	1880 (67.29)	1677 (63.28)	
Past year in-person visit(s) with health care clinician				
0	1146 (21.05)	589 (21.08)	557 (21.02)	.95
≥1	4298 (78.95)	2205 (78.92)	2093 (78.98)	
Ever tested positive for COVID-19				
Yes	1439 (26.43)	762 (27.27)	677 (25.54)	.15
No	4005 (73.57)	2032 (72.73)	1973 (74.46)	
BMI				
18.5 to <25	1590 (29.21)	790 (28.28)	800 (30.19)	.15
<18.5 or ≥25	3854 (70.79)	2004 (71.72)	1850 (69.81)	
Physical activity				
≥150 Minutes per week	3494 (64.18)	1829 (65.46)	1665 (62.83)	.04^b^
<150 Minutes per week	1950 (35.82)	965 (34.54)	985 (37.17)	
Past month cigarette smoking^d^				
Yes	1601 (29.41)	1005 (35.97)	596 (22.49)	<.001^b^
No	3843 (70.59)	1789 (64.03)	2054 (77.51)	
Past month e-cigarette use^d^				
Yes	918 (16.86)	557 (19.94)	361 (13.62)	<.001^b^
No	4526 (83.14)	2237 (80.06)	2289 (86.38)	
Alcohol misuse^d^				
Yes	350 (6.43)	174 (6.23)	176 (6.64)	.38
No	4848 (89.05)	2484 (88.90)	2364 (89.21)	
Not applicable, age <21 y	246 (4.52)	136 (4.87)	110 (4.15)	
Past month marijuana or cannabis use^d^				
Yes	1350 (24.80)	819 (29.31)	531 (20.04)	<.001^b^
No	4094 (75.20)	1975 (70.69)	2119 (79.96)	
Have internet access				
Yes	5325 (97.81)	2724 (97.49)	2601 (98.15)	.09
No	119 (2.19)	70 (2.51)	49 (1.85)	
Digital health literacy, mean (SE)^e^				
Using technology to process health information	2.91 (0.01)	2.94 (0.01)	2.89 (0.01)	<.001^b^
Understanding of health concepts and language	2.99 (0.01)	3.00 (0.01)	2.98 (0.01)	.04^b^
Ability to actively engage with digital services	2.95 (0.01)	2.98 (0.01)	2.93 (0.01)	.001^b^
Feel safe and in control	2.83 (0.01)	2.87 (0.01)	2.79 (0.01)	<.001^b^
Motivated to engage with digital services	2.89 (0.01)	2.92 (0.01)	2.86 (0.01)	<.001^b^
Access to digital services that work	2.93 (0.01)	2.94 (0.01)	2.91 (0.01)	.02^b^
Digital services that suit individual needs	2.80 (0.01)	2.86 (0.01)	2.75 (0.01)	<.001^b^
Outcome 1: telehealth use				
≥1	2754 (50.59)	1505 (53.87)	1249 (47.13)	<.001^b^
0	2690 (49.41)	1289 (46.13)	1401 (52.87)	
Outcome 2: telemedicine use				
≥1	1609 (29.56)	931 (33.32)	678 (25.58)	<.001^b^
0	3835 (70.44)	1863 (66.68)	1972 (74.42)	
Outcome 3: telemonitoring use				
Yes	854 (15.69)	551 (19.72)	303 (11.43)	<.001^b^
No	4590 (84.31)	2243 (80.28)	2347 (88.57)	

Abbreviations: BMI, body mass index (calculated as weight in kilograms divided by height in meters squared); MHSVI, Minority Health Social Vulnerability Index.

^a^ Most vulnerable counties fall in the top quartile of the MHSVI, whereas the least vulnerable counties fall in the bottom quartile of the MHSVI.

^b^ Statistical significance was set at *P* < .05.

^c^ Individuals who self-identified as homosexual, gay, lesbian, or bisexual were collapsed into the category not heterosexual.

^d^ Cigarette smoking, e-cigarette use, and marijuana use were coded as yes when participants self-reported use on 1 or more days in the past month; alcohol misuse was defined as 3 or more drinks per day for males and 2 or more drinks per day for females; otherwise alcohol misuse was coded as no.

^e^ Digital health literacy was measured using a 35-item, 1- to 4-point scale with higher scores indicating higher digital health literacy. Domain scores were averaged across items under each domain.

### Telehealth

Telehealth use was reportedly higher within the MHSVI most vulnerable counties (1505 of 2794 participants [53.87%] compared with least vulnerable counties (1249 of 2650 participants [47.13%]) (*P* < .001). Differences in telehealth use by MHSVI were not statistically significant when accounting for individual-level characteristics (Table 2). Overall, Black participants (adjusted odds ratio [aOR], 1.41; 95% CI, 1.17-1.70) and Hispanic participants (aOR, 1.41; 95% CI, 1.17-1.70) had higher odds of telehealth use compared with White participants (Black participants predictive margins [PM], 55%; 95% CI, 51%-58%; Hispanic participants PM, 55%; 95% CI, 51%-58%; White participants PM, 48%; 95% CI, 46%-49%). Participants who were English nonproficient (aOR, 1.54; 95% CI, 1.23-1.92) had higher odds of telehealth use compared with those who were proficient in English (PM, 58%; 95% CI, 54%-62% vs 49%; 95% CI, 48%-51%). Having fair or poor general health (aOR, 1.29; 95% CI, 1.09-1.52) was associated with higher odds of telehealth use compared with those with excellent to good health (PM, 54%; 95% CI, 51%-57% vs 49%; 95% CI, 47%-50%). In-person visits (aOR, 4.71; 95% CI, 3.93-5.63) were also associated with higher odds of telehealth use compared with those who did not have in-person visits (PM, 56%; 95% CI, 55%-58% vs 25%; 95% CI, 22%-28%). Having a BMI less than 18.5 or 25 or greater (aOR, 1.17; 95% CI, 1.02-1.36) or having reported cigarette smoking (aOR, 1.61; 95% CI, 1.37-1.90), e-cigarette use (aOR, 2.03; 95% CI, 1.65-2.50), or marijuana use (aOR, 1.31; 95% CI, 1.11-1.55) were associated with higher odds of telehealth use compared with individuals without these risk factors.

**Table 2.** Associations Between Sociodemographic, Health, and Technology Factors and Telehealth Use (N = 5444 Participants, Imputed Data)^a^

Factor	Overall (n = 5444)		MHSVI most vulnerable counties (n = 2794)		MHSVI least vulnerable counties (n = 2650)	
	PM (95% CI)	aOR (95% CI)	PM (95% CI)	aOR (95% CI)	PM (95% CI)	aOR (95% CI)
Age, y^b^						
18	0.52 (0.49-0.55)	0.99 (0.99-1.00)	0.58 (0.54-0.62)	0.99 (0.98-0.99)^c^	0.45 (0.41-0.50)	1.00 (0.99-1.00)
30	0.51 (0.49-0.53)		0.56 (0.53-0.59)		0.46 (0.43-0.49)	
45	0.50 (0.49-0.51)		0.53 (0.51-0.55)		0.47 (0.45-0.48)	
≥60	0.49 (0.47-0.51)		0.51 (0.48-0.53)		0.47 (0.45-0.50)	
Sex						
Female	0.51 (0.49-0.53)	1.09 (0.96-1.24)	0.54 (0.52-0.56)	1.09 (0.90-1.31)	0.48 (0.45-0.50)	1.09 (0.91-1.30)
Male	0.49 (0.47-0.51)	1 [Reference]	0.52 (0.50-0.55)	1 [Reference]	0.46 (0.43-0.48)	1 [Reference]
Sexual orientation						
Heterosexual	0.50 (0.48-0.51)	1 [Reference]	0.53 (0.51-0.55)	1 [Reference]	0.46 (0.44-0.48)	1 [Reference]
Not heterosexual^d^	0.54 (0.50-0.57)	1.22 (0.99-1.49)	0.54 (0.49-0.59)	1.05 (0.79-1.39)	0.54 (0.48-0.60)	1.47 (1.10-1.97)^c^
Race and ethnicity						
Hispanic or Latino	0.55 (0.51-0.58)	1.41 (1.17-1.70)^c^	0.56 (0.53-0.60)	1.33 (1.04-1.70)^c^	0.51 (0.45-0.58)	1.34 (0.97-1.85)
Non-Hispanic Black or African American	0.55 (0.51-0.58)	1.41 (1.17-1.70)^c^	0.55 (0.52-0.59)	1.27 (1.00-1.59)	0.59 (0.52-0.66)	1.99 (1.38-2.88)^c^
Non-Hispanic other^e^	0.53 (0.48-0.58)	1.29 (0.97-1.71)	0.57 (0.49-0.64)	1.37 (0.89-2.11)	0.49 (0.42-0.57)	1.21 (0.83-1.75)
Non-Hispanic White	0.48 (0.46-0.49)	1 [Reference]	0.51 (0.48-0.53)	1 [Reference]	0.45 (0.43-0.47)	1 [Reference]
Education						
Less than high school	0.47 (0.44-0.51)	1.07 (0.99-1.16)	0.48 (0.43-0.52)	1.18 (1.06-1.32)^c^	0.48 (0.43-0.53)	0.97 (0.86-1.08)
High school graduate	0.49 (0.47-0.51)		0.51 (0.48-0.53)		0.47 (0.44-0.51)	
Vocational school, some college	0.50 (0.49-0.51)		0.54 (0.52-0.56)		0.47 (0.45-0.49)	
College graduate or higher	0.52 (0.49-0.54)		0.57 (0.54-0.60)		0.46 (0.43-0.49)	
Income (in 2021)						
<$20 000	0.50 (0.48-0.53)	0.98 (0.92-1.05)	0.53 (0.50-0.56)	1.01 (0.91-1.11)	0.47 (0.44-0.51)	0.97 (0.89-1.07)
$20 000 to $49 999	0.50 (0.49-0.52)		0.53 (0.51-0.55)		0.47 (0.45-0.49)	
$50 000 to $74 999	0.50 (0.48-0.51)		0.54 (0.51-0.56)		0.47 (0.45-0.48)	
≥$75 000	0.50 (0.47-0.52)		0.54 (0.50-0.58)		0.46 (0.43-0.49)	
English proficiency						
Very well	0.49 (0.48-0.51)	1 [Reference]	0.52 (0.51-0.54)	1 [Reference]	0.46 (0.44-0.48)	1 [Reference]
Well, not well, not at all	0.58 (0.54-0.62)	1.54 (1.23-1.92)^c^	0.63 (0.58-0.68)	1.78 (1.30-2.42)^c^	0.52 (0.46-0.59)	1.34 (0.95-1.87)
Health insurance						
Insured	0.51 (0.50-0.52)	1 [Reference]	0.54 (0.52-0.56)	1 [Reference]	0.47 (0.45-0.49)	1 [Reference]
Uninsured	0.42 (0.38-0.46)	0.64 (0.52-0.79)^c^	0.47 (0.42-0.52)	0.67 (0.52-0.87)^c^	0.38 (0.31-0.45)	0.62 (0.43-0.89)^c^
General health						
Excellent, very good, good	0.49 (0.47-0.50)	1 [Reference]	0.52 (0.50-0.54)	1 [Reference]	0.46 (0.43-0.48)	1 [Reference]
Fair, poor	0.54 (0.51-0.57)	1.29 (1.09-1.52)^c^	0.58 (0.54-0.61)	1.38 (1.09-1.73)^c^	0.51 (0.46-0.55)	1.26 (1.00-1.60)
Mental health						
Excellent, very good, good	0.49 (0.48-0.51)	1 [Reference]	0.53 (0.51-0.56)	1 [Reference]	0.45 (0.43-0.48)	1 [Reference]
Fair, poor	0.52 (0.49-0.55)	1.13 (0.97-1.32)	0.53 (0.50-0.57)	0.99 (0.80-1.24)	0.51 (0.47-0.55)	1.29 (1.03-1.61)^c^
Having a primary care clinician						
Yes	0.54 (0.52-0.56)	1 [Reference]	0.58 (0.55-0.61)	1 [Reference]	0.50 (0.47-0.52)	1 [Reference]
No	0.46 (0.44-0.48)	0.68 (0.59-0.78)^c^	0.49 (0.46-0.52)	0.61 (0.50-0.75)^c^	0.43 (0.40-0.46)	0.74 (0.61-0.89)^c^
Presence of underlying medical conditions						
Yes	0.51 (0.49-0.54)	1.10 (0.95-1.28)	0.53 (0.49-0.56)	0.93 (0.75-1.15)	0.50 (0.46-0.53)	1.27 (1.03-1.55)
No	0.49 (0.48-0.51)	1 [Reference]	0.54 (0.51-0.56)	1 [Reference]	0.45 (0.42-0.47)	1 [Reference]
Past year in-person visit(s) with health care clinician						
0	0.25 (0.22-0.28)	1 [Reference]	0.24 (0.21-0.28)	1 [Reference]	0.26 (0.22-0.30)	1 [Reference]
≥1	0.56 (0.55-0.58)	4.71 (3.93-5.63)^c^	0.61 (0.59-0.63)	6.18 (4.79-7.96)^c^	0.52 (0.50-0.54)	3.58 (2.77-4.63)^c^
Ever tested positive for COVID-19						
Yes	0.55 (0.53-0.58)	1.43 (1.25-1.64)^c^	0.59 (0.55-0.62)	1.46 (1.20-1.78)^c^	0.52 (0.49-0.56)	1.44 (1.18-1.76)^c^
No	0.48 (0.47-0.50)	1 [Reference]	0.51 (0.49-0.53)	1 [Reference]	0.45 (0.42-0.47)	1 [Reference]
BMI						
18 to <25	0.48 (0.45-0.50)	1 [Reference]	0.49 (0.45-0.52)	1 [Reference]	0.47 (0.44-0.51)	1 [Reference]
<18.5 or ≥25	0.51 (0.49-0.53)	1.17 (1.02-1.36)^c^	0.55 (0.53-0.57)	1.39 (1.13-1.72)^c^	0.46 (0.44-0.49)	0.97 (0.79-1.18)
Physical activity						
≥150 Minutes per week	0.52 (0.50-0.53)	1 [Reference]	0.55 (0.53-0.58)	1 [Reference]	0.47 (0.45-0.50)	1 [Reference]
<150 Minutes per week	0.47 (0.45-0.50)	0.81 (0.71-0.92)^c^	0.50 (0.47-0.53)	0.74 (0.61-0.90)^c^	0.45 (0.42-0.48)	0.89 (0.74-1.07)
Past month cigarette smoking^f^						
Yes	0.57 (0.54-0.60)	1.61 (1.37-1.90)^c^	0.61 (0.58-0.64)	1.82 (1.45-2.28)^c^	0.53 (0.48-0.57)	1.46 (1.14-1.87)^c^
No	0.47 (0.46-0.49)	1 [Reference]	0.50 (0.47-0.52)	1 [Reference]	0.45 (0.43-0.47)	1 [Reference]
Past month e-cigarette use^f^						
Yes	0.62 (0.58-0.66)	2.03 (1.65-2.50)^c^	0.63 (0.58-0.67)	1.85 (1.39-2.45)^c^	0.60 (0.55-0.66)	2.13 (1.56-2.91)^c^
No	0.48 (0.46-0.49)	1 [Reference]	0.51 (0.49-0.53)	1 [Reference]	0.45 (0.43-0.47)	1 [Reference]
Alcohol misuse^f^						
Yes	0.48 (0.43-0.53)	0.89 (0.69-1.15)	0.50 (0.43-0.56)	0.81 (0.56-1.18)	0.45 (0.39-0.52)	0.92 (0.65-1.30)
No	0.50 (0.49-0.52)	1 [Reference]	0.53 (0.52-0.55)	1 [Reference]	0.47 (0.45-0.49)	1 [Reference]
Not applicable, age <21 y	0.50 (0.44-0.56)	0.97 (0.71-1.32)	0.57 (0.49-0.65)	1.19 (0.76-1.88)	0.42 (0.33-0.50)	0.76 (0.49-1.18)
Past month marijuana or cannabis use^f^						
Yes	0.54 (0.51-0.57)	1.31 (1.11-1.55)^c^	0.58 (0.54-0.61)	1.36 (1.08-1.72)^c^	0.50 (0.46-0.55)	1.23 (0.96-1.57)
No	0.49 (0.47-0.50)	1 [Reference]	0.52 (0.50-0.54)	1 [Reference]	0.46 (0.44-0.48)	1 [Reference]
Have internet access						
Yes	0.50 (0.49-0.51)	1 [Reference]	0.53 (0.51-0.55)	1 [Reference]	0.47 (0.45-0.49)	1 [Reference]
No	0.54 (0.45-0.62)	1.22 (0.78-1.90)	0.59 (0.48-0.68)	1.32 (0.74-2.38)	0.47 (0.33-0.62)	1.03 (0.50-2.10)
Digital health literacy^b^						
Using technology to process health information	0.48 (0.46-0.50)	1.32 (1.06-1.65)^c^	0.51 (0.48-0.54)	1.35 (0.97-1.87)	0.45 (0.42-0.48)	1.27 (0.92-1.74)
	0.52 (0.50-0.54)		0.55 (0.52-0.57)		0.48 (0.45-0.51)	
Understanding of health concepts and language	0.50 (0.48-0.52)	1.01 (0.82-1.25)	0.54 (0.52-0.56)	0.88 (0.64-1.21)	0.46 (0.44-0.48)	1.22 (0.90-1.65)
	0.50 (0.49-0.52)		0.53 (0.51-0.55)		0.48 (0.45-0.50)	
Ability to actively engage with digital services	0.51 (0.49-0.53)	0.85 (0.71-1.03)	0.54 (0.52-0.57)	0.87 (0.66-1.15)	0.48 (0.45-0.50)	0.85 (0.65-1.11)
	0.49 (0.48-0.51)		0.53 (0.51-0.55)		0.46 (0.43-0.48)	
Feel safe and in control	0.51 (0.49-0.52)	0.87 (0.75-1.00)	0.55 (0.53-0.57)	0.74 (0.59-0.93)^c^	0.47 (0.45-0.49)	0.97 (0.79-1.18)
	0.49 (0.47-0.51)		0.52 (0.49-0.54)		0.46 (0.44-0.49)	
Motivated to engage with digital services	0.49 (0.47-0.50)	1.29 (1.03-1.62)^c^	0.51 (0.48-0.54)	1.39 (0.99-1.95)	0.46 (0.43-0.48)	1.21 (0.88-1.66)
	0.52 (0.50-0.54)		0.55 (0.53-0.58)		0.48 (0.45-0.51)	
Access to digital services that work	0.50 (0.48-0.52)	1.04 (0.82-1.30)	0.53 (0.50-0.55)	1.12 (0.79-1.57)	0.47 (0.44-0.49)	0.96 (0.70-1.32)
	0.50 (0.49-0.52)		0.54 (0.52-0.56)		0.46 (0.44-0.49)	
Digital services that suit individual needs	0.49 (0.47-0.50)	1.28 (1.06-1.55)^c^	0.52 (0.49-0.55)	1.22 (0.93-1.60)	0.45 (0.42-0.47)	1.39 (1.06-1.83)^c^
	0.51 (0.50-0.53)		0.54 (0.52-0.56)		0.48 (0.46-0.51)	
MHSVI^g^						
Most vulnerable counties	0.51 (0.49-0.53)	1.06 (0.93-1.21)	NA	NA	NA	NA
Least vulnerable counties	0.49 (0.48-0.51)	1 [Reference]	NA	NA	NA	NA

Abbreviations: aOR, adjusted odds ratio; BMI, body mass index (calculated as weight in kilograms divided by height in meters squared); MHSVI, Minority Health Social Vulnerability Index; PM, predictive marginals.

^a^ Logistic regression analysis modeled the probability of 1 = 1 or more times of telehealth use. Income and education were entered as ordinal variables; age and digital health literacy were entered as continuous variables; all other variables (eg, race and ethnicity, sex) were entered as categorical variables.

^b^ Predictive marginals for digital health literacy domains recorded for 25th and 75th percentile of the score and for top age of all 4 age brackets.

^c^ Statistically significant results did not include the null value of 1 in the confidence interval.

^d^ Individuals who self-identified as homosexual, gay, lesbian, or bisexual were collapsed into the category not heterosexual.

^e^ Non-Hispanic Asian, Native Hawaiian, Pacific Islander, American Indian, and Alaska Native individuals were collapsed into non-Hispanic other.

^f^ Cigarette smoking, e-cigarette use, and marijuana use were coded as yes when participants self-reported use on 1 or more days in the past month; alcohol misuse was defined as 3 or more drinks per day for males and 2 or more drinks per day for females; otherwise alcohol misuse was coded as no.

^g^ Most vulnerable counties fall in the top quartile of the MHSVI, whereas the least vulnerable counties fall in the bottom quartile of the MHSVI.

The odds of using telehealth increased with 1-unit increase in the score of 3 digital health literacy domains (ie, using technology to process health information: aOR, 1.32; 95% CI, 1.06-1.65; being motivated to engage with digital services: aOR, 1.29; 95% CI, 1.03-1.62; having access to digital services that suit individual needs: aOR, 1.28; 95% CI, 1.06-1.55). For example, 52% (95% CI, 50%-54%) of participants who scored in the 75th percentile on the domain of using technology to process health information reported telehealth use vs 48% (95% CI, 46%-50%) of those who scored in the 25th percentile.

Similar associations existed across the MHSVI most vulnerable counties and least vulnerable counties with few exceptions. In the most vulnerable counties, the odds of telehealth use increased with incremental increases in educational attainment (aOR, 1.18; 95% CI, 1.06-1.32) and with being Hispanic (aOR, 1.33; 95% CI, 1.04-1.70). In the least vulnerable counties, self-identifying as Black (aOR, 1.99; 95% CI, 1.38-2.88) or not heterosexual (aOR, 1.47; 95% CI, 1.10-1.97), and having fair or poor mental health (aOR, 1.29; 95% CI, 1.03-1.61), or underlying medical conditions (aOR, 1.27; 95% CI, 1.03-1.55) were associated with higher odds of telehealth use. Increases in feeling safe and in control digital health literacy domain among participants in the most vulnerable MHSVI counties were associated with lower odds of use (aOR, 0.74; 95% CI, 0.59-0.93), whereas increases in having access to digital services that suit individual needs among those in the least vulnerable counties were associated with higher odds of use (aOR, 1.39; 95% CI, 1.06-1.83).

### Telemedicine

Telemedicine use was reportedly higher within the most vulnerable counties (931 of 2794 participants [33.32%]) compared with the least vulnerable counties (678 of 2650 participants [25.58%]) (*P* < .001). When accounting for individual-level characteristics, differences by MHSVI were not statistically significant (Table 3). Race and ethnicity, language proficiency, having a PCC, in-person visits, risk factors (ie, physical activity, cigarette smoking, e-cigarette and marijuana use), and using technology to process health information showed associations with telemedicine use that were similar in significance and direction to those reported for telehealth use.

**Table 3.** Associations Between Sociodemographic, Health, and Technology Factors and Telemedicine Use (N = 5444 Participants, Imputed Data)^a^

Factor	Overall (n = 5444)		MHSVI most vulnerable counties (n = 2794)		MHSVI least vulnerable counties (n = 2650)	
	PM (95% CI)	aOR (95% CI)	PM (95% CI)	aOR (95% CI)	PM (95% CI)	aOR (95% CI)
Age, y^b^						
18	0.38 (0.35-0.41)	0.98 (0.97-0.98)^c^	0.43 (0.39-0.47)	0.97 (0.97-0.98)^c^	0.33 (0.29-0.37)	0.98 (0.97-0.99)^c^
30	0.34 (0.32-0.36)		0.38 (0.35-0.41)		0.29 (0.27-0.32)	
45	0.29 (0.27-0.30)		0.32 (0.30-0.34)		0.25 (0.24-0.27)	
60	0.24 (0.22-0.26)		0.26 (0.24-0.29)		0.21 (0.19-0.24)	
Sex						
Female	0.29 (0.27-0.31)	0.98 (0.85-1.13)	0.33 (0.30-0.35)	0.96 (0.79-1.16)	0.25 (0.23-0.27)	0.98 (0.80-1.21)
Male	0.29 (0.27-0.31)	1 [Reference]	0.33 (0.31-0.36)	1 [Reference]	0.25 (0.23-0.28)	1 [Reference]
Sexual orientation						
Heterosexual	0.29 (0.27-0.30)	1 [Reference]	0.33 (0.31-0.34)	1 [Reference]	0.24 (0.22-0.26)	1 [Reference]
Not heterosexual^d^	0.33 (0.29-0.36)	1.28 (1.04-1.57)^c^	0.34 (0.30-0.39)	1.09 (0.82-1.46)	0.32 (0.27-0.37)	1.57 (1.16-2.11)^c^
Race and ethnicity						
Hispanic or Latino	0.31 (0.29-0.34)	1.27 (1.04-1.54)^c^	0.34 (0.31-0.38)	1.24 (0.97-1.58)	0.28 (0.23-0.34)	1.28 (0.90-1.82)
Non-Hispanic Black or African American	0.34 (0.31-0.37)	1.44 (1.18-1.76)^c^	0.37 (0.33-0.40)	1.41 (1.11-1.80)^c^	0.31 (0.25-0.38)	1.49 (1.01-2.19)^c^
Non-Hispanic other^e^	0.26 (0.22-0.31)	0.93 (0.68-1.28)	0.29 (0.22-0.36)	0.90 (0.56-1.43)	0.24 (0.18-0.30)	0.94 (0.60-1.47)
Non-Hispanic White	0.27 (0.26-0.29)	1 [Reference]	0.31 (0.28-0.33)	1 [Reference]	0.24 (0.23-0.26)	1 [Reference]
Education						
Less than high school	0.25 (0.23-0.28)	1.14 (1.04-1.24)^c^	0.28 (0.24-0.32)	1.18 (1.05-1.33)^c^	0.23 (0.19-0.27)	1.08 (0.94-1.24)
High school graduate	0.27 (0.25-0.29)		0.31 (0.28-0.33)		0.24 (0.21-0.27)	
Vocational school, some college	0.29 (0.28-0.30)		0.33 (0.32-0.35)		0.25 (0.23-0.27)	
College graduate or higher	0.31 (0.29-0.33)		0.36 (0.33-0.39)		0.26 (0.24-0.29)	
Income (in 2021)						
<$20 000	0.30 (0.28-0.32)	0.96 (0.89-1.03)	0.34 (0.31-0.36)	0.95 (0.86-1.05)	0.26 (0.23-0.29)	0.97 (0.87-1.08)
$20 000 to $49 999	0.29 (0.28-0.31)		0.33 (0.31-0.35)		0.25 (0.23-0.28)	
$50 000 to $74 999	0.29 (0.27-0.30)		0.32 (0.30-0.34)		0.25 (0.23-0.27)	
≥$75 000	0.28 (0.26-0.30)		0.32 (0.28-0.35)		0.25 (0.22-0.27)	
English proficiency						
Very well	0.28 (0.27-0.30)	1 [Reference]	0.32 (0.30-0.34)	1 [Reference]	0.25 (0.23-0.26)	1 [Reference]
Well, not well, not at all	0.36 (0.33-0.40)	1.60 (1.28-1.99)^c^	0.42 (0.37-0.47)	1.79 (1.34-2.41)^c^	0.30 (0.25-0.36)	1.40 (0.99-1.98)
Health insurance						
Insured	0.29 (0.28-0.31)	1 [Reference]	0.33 (0.31-0.35)	1 [Reference]	0.25 (0.23-0.27)	1 [Reference]
Uninsured	0.27 (0.24-0.31)	0.86 (0.68-1.09)	0.30 (0.26-0.35)	0.83 (0.62-1.10)	0.25 (0.20-0.32)	1.02 (0.67-1.55)
General health						
Excellent, very good, good	0.29 (0.27-0.30)	1 [Reference]	0.32 (0.30-0.35)	1 [Reference]	0.25 (0.23-0.27)	1 [Reference]
Fair, poor	0.30 (0.27-0.32)	1.04 (0.86-1.25)	0.34 (0.30-0.37)	1.07 (0.83-1.38)	0.25 (0.22-0.29)	1.02 (0.77-1.35)
Mental health						
Excellent, very good, good	0.28 (0.27-0.30)	1 [Reference]	0.33 (0.31-0.35)	1 [Reference]	0.24 (0.22-0.26)	1 [Reference]
Fair, poor	0.31 (0.28-0.33)	1.13 (0.95-1.35)	0.33 (0.30-0.37)	1.03 (0.81-1.30)	0.28 (0.24-0.31)	1.27 (0.98-1.65)
Having a primary care clinician						
Yes	0.33 (0.31-0.35)	1 [Reference]	0.38 (0.35-0.40)	1 [Reference]	0.28 (0.26-0.31)	1 [Reference]
No	0.25 (0.24-0.27)	0.62 (0.54-0.72)^c^	0.29 (0.27-0.31)	0.58 (0.47-0.71)^c^	0.22 (0.20-0.24)	0.66 (0.53-0.82)^c^
Presence of underlying medical conditions						
Yes	0.28 (0.26-0.30)	0.93 (0.80-1.10)	0.31 (0.29-0.35)	0.88 (0.70-1.10)	0.25 (0.22-0.28)	0.98 (0.78-1.23)
No	0.29 (0.28-0.31)	1 [Reference]	0.34 (0.31-0.36)	1 [Reference]	0.25 (0.23-0.27)	1 [Reference]
Past year in-person visit(s) with health care clinician						
0	0.12 (0.10-0.15)	1 [Reference]	0.13 (0.10-0.16)	1 [Reference]	0.12 (0.09-0.16)	1 [Reference]
≥1	0.33 (0.32-0.34)	4.34 (3.42-5.51)^c^	0.37 (0.35-0.40)	5.54 (3.97-7.75)^c^	0.28 (0.26-0.30)	3.32 (2.35-4.69)^c^
Ever tested positive for COVID-19						
Yes	0.28 (0.26-0.31)	0.93 (0.80-1.09)	0.32 (0.29-0.35)	0.91 (0.74-1.12)	0.25 (0.22-0.28)	0.97 (0.77-1.22)
No	0.29 (0.28-0.31)	1 [Reference]	0.33 (0.31-0.35)	1 [Reference]	0.25 (0.23-0.27)	1 [Reference]
BMI						
18.5 to <25	0.27 (0.25-0.29)	1 [Reference]	0.30 (0.27-0.33)	1 [Reference]	0.24 (0.22-0.27)	1 [Reference]
<18.5 or ≥25	0.30 (0.28-0.31)	1.17 (1.00-1.36)	0.34 (0.32-0.36)	1.24 (1.00-1.54)	0.25 (0.23-0.27)	1.06 (0.84-1.33)
Physical activity						
≥150 Minutes per week	0.31 (0.29-0.32)	1 [Reference]	0.35 (0.33-0.37)	1 [Reference]	0.27 (0.25-0.29)	1 [Reference]
<150 Minutes per week	0.25 (0.23-0.27)	0.70 (0.60-0.82)^c^	0.29 (0.26-0.32)	0.69 (0.56-0.86)^c^	0.22 (0.19-0.25)	0.73 (0.58-0.91)^c^
Past month cigarette smoking^f^						
Yes	0.36 (0.33-0.38)	1.73 (1.46-2.05)^c^	0.39 (0.36-0.42)	1.71 (1.37-2.15)^c^	0.32 (0.29-0.36)	1.77 (1.38-2.28)^c^
No	0.26 (0.25-0.28)	1 [Reference]	0.29 (0.27-0.32)	1 [Reference]	0.23 (0.21-0.25)	1 [Reference]
Past month e-cigarette use^f^						
Yes	0.39 (0.36-0.42)	1.95 (1.62-2.36)^c^	0.41 (0.37-0.46)	1.83 (1.42-2.36)^c^	0.35 (0.30-0.40)	2.02 (1.52-2.70)^c^
No	0.27 (0.25-0.28)	1 [Reference]	0.31 (0.29-0.33)	1 [Reference]	0.23 (0.21-0.25)	1 [Reference]
Alcohol misuse^f^						
Yes	0.28 (0.24-0.32)	0.93 (0.70-1.22)	0.32 (0.27-0.39)	0.97 (0.67-1.40)	0.23 (0.18-0.30)	0.88 (0.58-1.33)
No	0.29 (0.28-0.30)	1 [Reference]	0.33 (0.31-0.35)	1 [Reference]	0.25 (0.23-0.27)	1 [Reference]
Not applicable, age <21 y	0.31 (0.26-0.36)	1.11 (0.81-1.52)	0.35 (0.28-0.43)	1.13 (0.72-1.77)	0.27 (0.20-0.34)	1.10 (0.70-1.71)
Past month marijuana or cannabis use^f^						
Yes	0.36 (0.33. 0.39)	1.71 (1.45-2.03)^c^	0.41 (0.37-0.44)	1.88 (1.50-2.37)^c^	0.30 (0.26-0.34)	1.50 (1.16-1.94)^c^
No	0.27 (0.25-0.28)	1 [Reference]	0.29 (0.27-0.31)	1 [Reference]	0.24 (0.22-0.26)	1 [Reference]
Have internet access						
Yes	0.29 (0.28-0.30)	1 [Reference]	0.33 (0.31-0.35)	1 [Reference]	0.25 (0.23-0.27)	1 [Reference]
No	0.32 (0.25-0.40)	1.19 (0.74-1.91)	0.34 (0.25-0.44)	1.06 (0.59-1.90)	0.30 (0.19-0.45)	1.40 (0.64-3.06)
Digital health literacy^b^						
Using technology to process health information	0.26 (0.24-0.28)	1.72 (1.34-2.22)^c^	0.30 (0.27-0.32)	1.63 (1.16-2.29)^c^	0.22 (0.19-0.24)	1.81 (1.24-2.65)^c^
	0.31 (0.29-0.33)		0.34 (0.32-0.37)		0.27 (0.25-0.30)	
Understanding of health concepts and language	0.30 (0.28-0.31)	0.83 (0.66-1.06)	0.33 (0.31-0.36)	0.88 (0.63-1.22)	0.26 (0.24-0.28)	0.83 (0.58-1.17)
	0.29 (0.27-0.30)		0.33 (0.31-0.34)		0.25 (0.23-0.27)	
Ability to actively engage with digital services	0.31 (0.29-0.33)	0.75 (0.61-0.92)^c^	0.35 (0.32-0.38)	0.76 (0.57-1.01)	0.27 (0.25-0.30)	0.72 (0.53-0.98)^c^
	0.28 (0.27-0.30)		0.32 (0.30-0.34)		0.24 (0.23-0.26)	
Feel safe and in control	0.29 (0.28-0.30)	1.01 (0.86-1.19)	0.33 (0.31-0.35)	1.02 (0.80-1.29)	0.25 (0.23-0.27)	0.98 (0.77-1.24)
	0.29 (0.28-0.31)		0.33 (0.31-0.35)		0.25 (0.23-0.27)	
Motivated to engage with digital services	0.27 (0.25-0.29)	1.37 (1.07-1.76)^c^	0.31 (0.29-0.34)	1.26 (0.89-1.79)	0.23 (0.21-0.26)	1.47 (1.02-2.11)^c^
	0.30 (0.29-0.32)		0.34 (0.32-0.36)		0.26 (0.24-0.29)	
Access to digital services that work	0.31 (0.29-0.33)	0.71 (0.55-0.92)^c^	0.34 (0.32-0.37)	0.77 (0.54-1.11)	0.27 (0.25-0.30)	0.68 (0.47-0.98)^c^
	0.28 (0.27-0.30)		0.32 (0.30-0.34)		0.24 (0.22-0.26)	
Digital services that suit individual needs	0.27 (0.25-0.28)	1.45 (1.17-1.78)^c^	0.31 (0.29-0.34)	1.24 (0.94-1.63)	0.22 (0.20-0.24)	1.83 (1.33-2.52)^c^
	0.30 (0.28-0.31)		0.33 (0.31-0.35)		0.27 (0.25-0.29)	
MHSVI^g^						
Most vulnerable counties	0.29 (0.28-0.31)	1.04 (0.90-1.21)	NA	NA	NA	NA
Least vulnerable counties	0.29 (0.27-0.30)	1 [Reference]	NA	NA	NA	NA

Abbreviations: aOR, adjusted odds ratio; BMI, body mass index (calculated as weight in kilograms divided by height in meters squared); MHSVI, Minority Health Social Vulnerability Index; PM, predictive marginals.

^a^ Logistic regression analysis modeled the probability of 1 = 1 or more times of telemedicine use. Income and education were entered as ordinal variables; age and digital literacy were entered as continuous variables; all other variables (eg, race and ethnicity, sex) were entered as categorical variables.

^b^ Predictive marginals for digital health literacy domains recorded for 25th and 75th percentile of the score and for top age of all 4 age brackets.

^c^ Statistically significant results did not include the null value of 1 in the confidence interval.

^d^ Individuals who self-identified as homosexual, gay, lesbian, or bisexual were collapsed into the category not heterosexual.

^e^ Non-Hispanic Asian, Native Hawaiian, Pacific Islander, American Indian, and Alaska Native individuals were collapsed into non-Hispanic other.

^f^ Cigarette smoking, e-cigarette use, and marijuana use were coded as yes when participants self-reported use on 1 or more days in the past month; alcohol misuse was defined as 3 or more drinks per day for males and 2 or more drinks per day for females; otherwise alcohol misuse was coded as no.

^g^ Most vulnerable counties fall in the top quartile of the MHSVI, whereas the least vulnerable counties fall in the bottom quartile of the MHSVI.

Associations between telemedicine use and education, sexual orientation, and digital health literacy differed across MHSVI. Incremental increases in educational attainment were associated with higher odds of telemedicine use overall (aOR, 1.14; 95% CI, 1.04-1.24) and in the most vulnerable counties (aOR, 1.18; 95% CI, 1.05-1.33). Identifying as nonheterosexual was associated with higher odds of telemedicine use overall (aOR, 1.28; 95% CI, 1.04-1.57) and in the least vulnerable counties (aOR, 1.57; 95% CI, 1.16-2.11). The ability to actively engage with digital services (aOR, 0.75; 95% CI, 0.61-0.92; aOR, 0.72; 95% CI, 0.53-0.98) and access to digital devices that work (aOR, 0.71; 95% CI, 0.55-0.92; aOR, 0.68; 95% CI, 0.47-0.98) were associated with lower odds of telemedicine use overall and in least vulnerable counties. Conversely, motivation to engage in digital services overall (aOR, 1.37; 95% CI, 1.07-1.76) and in the least vulnerable counties (aOR, 1.47; 95% CI, 1.02-2.11) and access to digital services that suit individual needs overall (aOR, 1.45; 95% CI, 1.17-1.78) and in the least vulnerable counties (aOR, 1.83; 95% CI, 1.33-2.52) were associated with higher odds of telemedicine use.

### Telemonitoring

Telemonitoring use was reportedly higher within the most vulnerable counties (551 of 2794 participants [19.72%]) compared with the least vulnerable counties (303 of 2650 participants [11.43%]) (*P* < .001). These differences remained statistically significant after adjusting for individual-level characteristics (aOR, 1.42; 95% CI, 1.18-1.70) (Table 4). Race and ethnicity, language proficiency, general and mental health, having a PCC, in-person visits, and risk factors (ie, physical activity, cigarette smoking, e-cigarette and marijuana use) showed associations with telemonitoring use that were similar in significance and direction to those reported for telehealth and telemedicine use. Overall and within both MHSVI strata, the ability to actively engage with digital services was associated with lower odds of telemonitoring use, whereas access to digital services that suit individual needs was associated with higher use odds. Incremental increases in motivation to engage with digital services were associated with increased odds of telemonitoring use overall (aOR, 1.47; 95% CI, 1.06-2.03), whereas feeling safe and in control was associated with increased odds of telemonitoring use in the most vulnerable counties only (aOR, 1.34; 95% CI, 1.01-1.78). Results from the multiple imputation and complete case analyses were largely similar (eTables 4 to 6 in Supplement 1).

**Table 4.** Associations Between Sociodemographic, Health, and Technology Factors and Telemonitoring Use (N = 5444 Participants, Imputed Data)^a^

Factor	Overall (n = 5444)		MHSVI most vulnerable counties (n = 2794)		MHSVI least vulnerable counties (n = 2650)	
	PM (95% CI)	aOR (95% CI)	PM (95% CI)	aOR (95% CI)	PM (95% CI)	aOR (95% CI)
Age, y^b^						
18	0.19 (0.17-0.22)	0.98 (0.97-0.99)^c^	0.26 (0.22-0.30)	0.98 (0.97-0.98)^c^	0.13 (0.10-0.16)	0.99 (0.98-1.00)
30	0.17 (0.16-0.19)		0.22 (0.20-0.24)		0.12 (0.10-0.14)	
45	0.15 (0.14-0.16)		0.18 (0.17-0.20)		0.11 (0.10-0.12)	
60	0.13 (0.11-0.14)		0.15 (0.13-0.17)		0.10 (0.08-0.12)	
Sex						
Female	0.15 (0.14-0.16)	0.91 (0.76-1.08)	0.20 (0.18-0.21)	1.06 (0.84-1.33)	0.10 (0.08-0.11)	0.71 (0.54-0.94)^c^
Male	0.16 (0.14-0.17)	1 [Reference]	0.19 (0.17-0.21)	1 [Reference]	0.12 (0.11-0.14)	1 [Reference]
Sexual orientation						
Heterosexual	0.15 (0.14-0.16)	1 [Reference]	0.19 (0.18-0.21)	1 [Reference]	0.10 (0.09-0.12)	1 [Reference]
Not heterosexual^d^	0.16 (0.14-0.19)	1.12 (0.86-1.45)	0.18 (0.15-0.22)	0.92 (0.66-1.28)	0.14 (0.11-0.19)	1.54 (1.02-2.31)^c^
Race and ethnicity						
Hispanic or Latino	0.18 (0.16-0.20)	1.46 (1.16-1.84)^c^	0.22 (0.19-0.25)	1.55 (1.17-2.05)^c^	0.12 (0.08-0.16)	1.15 (0.74-1.80)
Non-Hispanic Black or African American	0.17 (0.15-0.20)	1.40 (1.11-1.78)^c^	0.21 (0.18-0.24)	1.37 (1.03-1.82)^c^	0.14 (0.10-0.20)	1.49 (0.91-2.45)
Non-Hispanic Other^e^	0.16 (0.12-0.20)	1.24 (0.86-1.78)	0.20 (0.15-0.27)	1.36 (0.84-2.19)	0.11 (0.07-0.17)	1.10 (0.61-2.00)
Non-Hispanic White	0.14 (0.12-0.15)	1 [Reference]	0.17 (0.15-0.19)	1 [Reference]	0.11 (0.09-0.12)	1 [Reference]
Education						
Less than high school	0.15 (0.13-0.17)	1.01 (0.90-1.12)	0.18 (0.15-0.22)	1.04 (0.90-1.20)	0.12 (0.09-0.15)	0.96 (0.80-1.15)
High school graduate	0.15 (0.14-0.17)		0.19 (0.17-0.21)		0.11 (0.09-0.13)	
Vocational school, some college	0.15 (0.14-0.16)		0.19 (0.18-0.21)		0.11 (0.10-0.12)	
College graduate or higher	0.15 (0.14-0.17)		0.20 (0.17-0.23)		0.11 (0.09-0.13)	
Income (in 2021)						
<$20 000	0.15 (0.13-0.16)	1.04 (0.95-1.13)	0.19 (0.16-0.21)	1.04 (0.93-1.17)	0.10 (0.08-0.13)	1.05 (0.92-1.21)
$20 000 to $49 999	0.15 (0.14-0.16)		0.19 (0.18-0.21)		0.11 (0.09-0.12)	
$50 000 to $74 999	0.15 (0.14-0.17)		0.20 (0.18-0.22)		0.11 (0.10-0.12)	
≥$75 000	0.16 (0.14-0.18)		0.20 (0.17-0.24)		0.12 (0.10-0.14)	
English proficiency						
Very well	0.15 (0.14-0.16)	1 [Reference]	0.19 (0.17-0.20)	1 [Reference]	0.10 (0.09-0.12)	1 [Reference]
Well, not well, not at all	0.21 (0.18-0.25)	1.74 (1.34-2.26)^c^	0.25 (0.21-0.30)	1.61 (1.16-2.23)^c^	0.18 (0.13-0.24)	2.11 (1.35-3.29)^c^
Health insurance						
Insured	0.15 (0.14-0.16)	1 [Reference]	0.20 (0.18-0.21)	1 [Reference]	0.11 (0.10-0.12)	1 [Reference]
Uninsured	0.14 (0.11-0.17)	0.87 (0.65-1.17)	0.16 (0.13-0.20)	0.74 (0.53-1.05)	0.15 (0.10-0.21)	1.50 (0.88-2.56)
General health						
Excellent, very good, good	0.14 (0.13-0.16)	1 [Reference]	0.18 (0.17-0.20)	1 [Reference]	0.10 (0.09-0.12)	1 [Reference]
Fair, poor	0.18 (0.16-0.20)	1.35 (1.07-1.69)^c^	0.22 (0.19-0.26)	1.34 (0.99-1.80)	0.14 (0.11-0.17)	1.45 (1.01-2.09)^c^
Mental health						
Excellent, very good, good	0.16 (0.15-0.18)	1 [Reference]	0.21 (0.19-0.23)	1 [Reference]	0.12 (0.10-0.13)	1 [Reference]
Fair, poor	0.12 (0.11-0.14)	0.66 (0.53-0.82)^c^	0.15 (0.13-0.18)	0.60 (0.45-0.80)^c^	0.09 (0.07-0.12)	0.76 (0.53-1.09)
Having a primary care clinician						
Yes	0.18 (0.16-0.19)	1 [Reference]	0.23 (0.21-0.25)	1 [Reference]	0.13 (0.11-0.14)	1 [Reference]
No	0.13 (0.12-0.14)	0.62 (0.52-0.75)^c^	0.16 (0.14-0.18)	0.58 (0.46-0.74)^c^	0.09 (0.08-0.11)	0.67 (0.50-0.90)^c^
Presence of underlying medical conditions						
Yes	0.15 (0.13-0.16)	0.90 (0.74-1.10)	0.18 (0.15-0.21)	0.83 (0.64-1.09)	0.11 (0.09-0.13)	0.98 (0.73-1.33)
No	0.16 (0.14-0.17)	1 [Reference]	0.20 (0.18-0.22)	1 [Reference]	0.11 (0.10-0.13)	1 [Reference]
Past year in-person visit(s) with health care clinician						
0	0.07 (0.05-0.09)	1 [Reference]	0.08 (0.06-0.11)	1 [Reference]	0.06 (0.04-0.09)	1 [Reference]
≥1	0.17 (0.16-0.18)	3.17 (2.32-4.32)^c^	0.21 (0.20-0.23)	3.75 (2.49-5.65)^c^	0.12 (0.11-0.13)	2.40 (1.50-3.86)^c^
Ever tested positive for COVID-19						
Yes	0.17 (0.15-0.19)	1.26 (1.05-1.51)^c^	0.21 (0.18-0.23)	1.15 (0.90-1.46)	0.14 (0.11-0.16)	1.49 (1.12-1.99)^c^
No	0.14 (0.13-0.16)	1 [Reference]	0.19 (0.17-0.20)	1 [Reference]	0.10 (0.09-0.11)	1 [Reference]
BMI						
18.5 to <25	0.15 (0.13-0.17)	1 [Reference]	0.20 (0.17-0.22)	1 [Reference]	0.10 (0.08-0.13)	1 [Reference]
<18.5 or ≥25	0.15 (0.14-0.17)	1.04 (0.86-1.27)	0.19 (0.17-0.21)	0.96 (0.74-1.25)	0.11 (0.10-0.13)	1.11 (0.81-1.53)
Physical activity						
≥150 Minutes per week	0.17 (0.16-0.18)	1 [Reference]	0.21 (0.19-0.23)	1 [Reference]	0.13 (0.11-0.14)	1 [Reference]
<150 Minutes per week	0.11 (0.10-0.13)	0.55 (0.45-0.68)^c^	0.15 (0.13-0.18)	0.62 (0.47-0.80)^c^	0.07 (0.05-0.09)	0.47 (0.33-0.66)^c^
Past month cigarette smoking^f^						
Yes	0.18 (0.16-0.20)	1.45 (1.18-1.77)^c^	0.22 (0.19-0.24)	1.37 (1.05-1.77)^c^	0.14 (0.12-0.17)	1.64 (1.18-2.28)^c^
No	0.14 (0.12-0.15)	1 [Reference]	0.17 (0.16-0.20)	1 [Reference]	0.10 (0.08-0.11)	1 [Reference]
Past month e-cigarette use^f^						
Yes	0.22 (0.20-0.25)	2.15 (1.74-2.66)^c^	0.26 (0.23-0.30)	1.98 (1.52-2.60)^c^	0.18 (0.14-0.22)	2.28 (1.60-3.24)^c^
No	0.13 (0.12-0.14)	1 [Reference]	0.17 (0.15-0.18)	1 [Reference]	0.09 (0.08-0.11)	1 [Reference]
Alcohol misuse^f^						
Yes	0.16 (0.12-0.19)	1.03 (0.75-1.43)	0.19 (0.14-0.25)	0.96 (0.62-1.49)	0.11 (0.08-0.16)	1.04 (0.64-1.71)
No	0.15 (0.14-0.16)	1 [Reference]	0.19 (0.18-0.21)	1 [Reference]	0.11 (0.10-0.12)	1 [Reference]
Not applicable, age <21 y	0.16 (0.12-0.20)	1.05 (0.72-1.54)	0.20 (0.15-0.27)	1.05 (0.65-1.71)	0.11 (0.07-0.18)	1.04 (0.55-1.94)
Past month marijuana or cannabis use^f^						
Yes	0.19 (0.17-0.21)	1.54 (1.27-1.88)^c^	0.23 (0.20-0.26)	1.59 (1.24-2.05)^c^	0.13 (0.11-0.16)	1.45 (1.04-2.00)^c^
No	0.14 (0.12-0.15)	1 [Reference]	0.17 (0.15-0.19)	1 [Reference]	0.10 (0.09-0.11)	1 [Reference]
Have internet access						
Yes	0.15 (0.14-0.16)	1 [Reference]	0.19 (0.18-0.21)	1 [Reference]	0.11 (0.10-0.12)	1 [Reference]
No	0.16 (0.11-0.24)	1.09 (0.60-1.96)	0.21 (0.13-0.31)	1.11 (0.57-2.16)	0.10 (0.04-0.22)	0.93 (0.31-2.71)
Digital health literacy^b^						
Using technology to process health information	0.15 (0.13-0.16)	1.14 (0.84-1.55)	0.18 (0.16-0.21)	1.17 (0.79-1.74)	0.11 (0.09-0.13)	1.02 (0.61-1.68)
	0.15 (0.14-0.17)		0.20 (0.18-0.21)		0.11 (0.10-0.12)	
Understanding of health concepts and language	0.15 (0.14-0.17)	0.91 (0.68-1.23)	0.20 (0.18-0.22)	0.90 (0.63-1.31)	0.11 (0.09-0.13)	0.96 (0.58-1.60)
	0.15 (0.14-0.16)		0.19 (0.18-0.21)		0.11 (0.10-0.12)	
Ability to actively engage with digital services	0.18 (0.16-0.20)	0.57 (0.44-0.73)^c^	0.23 (0.21-0.26)	0.52 (0.37-0.71)^c^	0.13 (0.11-0.15)	0.62 (0.40-0.94)^c^
	0.15 (0.14-0.16)		0.18 (0.17-0.20)		0.10 (0.09-0.12)	
Feel safe and in control	0.15 (0.13-0.16)	1.17 (0.96-1.43)	0.18 (0.16-0.20)	1.34 (1.01-1.78)^c^	0.11 (0.10-0.12)	0.99 (0.74-1.33)
	0.16 (0.15-0.17)		0.20 (0.18-0.22)		0.11 (0.10-0.12)	
Motivated to engage with digital services	0.13 (0.12-0.15)	1.47 (1.06-2.03)^c^	0.17 (0.15-0.20)	1.49 (0.98-2.25)	0.10 (0.08-0.12)	1.46 (0.87-2.45)
	0.16 (0.15-0.17)		0.20 (0.18-0.22)		0.12 (0.10-0.13)	
Access to digital services that work	0.15 (0.13-0.16)	1.15 (0.82-1.59)	0.19 (0.16-0.22)	1.07 (0.68-1.66)	0.10 (0.08-0.12)	1.27 (0.76-2.14)
	0.15 (0.14-0.16)		0.19 (0.18-0.21)		0.11 (0.10-0.13)	
Digital services that suit individual needs	0.13 (0.12-0.14)	1.60 (1.24-2.08)^c^	0.17 (0.15-0.19)	1.45 (1.05-2.00)^c^	0.09 (0.07-0.10)	2.05 (1.31-3.21)^c^
	0.15 (0.14-0.16)		0.19 (0.18-0.21)		0.11 (0.10-0.13)	
MHSVI^g^						
Most vulnerable counties	0.17 (0.16-0.18)	1.42 (1.18-1.70)^c^	NA	NA	NA	NA
Least vulnerable counties	0.13 (0.12-0.15)	1 [Reference]	NA	NA	NA	NA

Abbreviations: aOR, adjusted odds ratio; BMI, body mass index (calculated as weight in kilograms divided by height in meters squared); MHSVI, Minority Health Social Vulnerability Index; PM, predictive marginals.

^a^ Logistic regression analysis modeled the probability of 1 = telemonitoring use. Income and education were entered as ordinal variables; age and digital literacy were entered as continuous variables; all other variables (eg, race and ethnicity, sex) were entered as categorical variables.

^b^ Predictive marginals for digital health literacy domains recorded for 25th and 75th percentile of the score and for top age of all 4 age brackets.

^c^ Statistically significant results did not include the null value of 1 in the confidence interval.

^d^ Individuals who self-identified as homosexual, gay, lesbian, or bisexual were collapsed into the category not heterosexual.

^e^ Non-Hispanic Asian, Native Hawaiian, Pacific Islander, American Indian, and Alaska Native individuals were collapsed into non-Hispanic other.

^f^ Cigarette smoking, e-cigarette use, and marijuana use were coded as yes when participants self-reported use on 1 or more days in the past month; alcohol misuse was defined as 3 or more drinks per day for males and 2 or more drinks per day for females; otherwise alcohol misuse was coded as no.

^g^ Most vulnerable counties fall in the top quartile of the MHSVI, whereas the least vulnerable counties fall in the bottom quartile of the MHSVI.

## Discussion

The findings of this cross-sectional study demonstrated the interplay between individual-level characteristics and county-level indicators of disadvantage on digital health care use. First, telehealth use was reportedly higher than telemedicine and telemonitoring use. Second, although digital health care use was associated with well-documented factors of disparate access to in-person health care, some traditionally underserved participants had higher odds of use. Third, although county-level social vulnerability was only significantly associated with telemonitoring use, several individual-level characteristics varied in their associations with digital health care use across MHSVI strata. Within the most vulnerable counties, higher educational attainment was associated with increased odds of digital health care use, whereas self-identifying as not heterosexual and having poor mental health were associated with increased odds of use only in the least vulnerable counties. These findings suggest that some disparities traditionally associated with health care access carried over to digital health care.^36,37^ Increases in digital health care use among traditionally underserved participants reflected promising trends where digital health care can close gaps in health care access.^38^ Given the prominence of digital health care,^38^ these findings suggest the need to ensure continued digital health care access by replacing expiring regulatory policies brought about by the COVID-19 pandemic, addressing individual-level factors associated with limited use, and reducing county-level social vulnerability.

This study documented distinct participant characteristics associated with the use of telehealth, telemedicine, and telemonitoring. Telehealth has been used as an umbrella term encompassing remote health care services.^39^ However, each service offers different types of care with unique requisites for their provision. For instance, telemonitoring requires the use of digital devices (eg, blood glucose monitors), integration with electronic health records, and trained staff, among others.^40,41,42^ As of 2021, 72% of US hospitals provided at least 1 digital health care service with 55% offering telemedicine consultations and 29% offering remote patient monitoring.^43^ Efforts are needed to make digital health care services uniformly available across hospitals and to promote awareness of their availability.^44^

Consistent with prior literature,^17,18,19,23,45^ not having health insurance or a PCC were associated with lower odds of using digital health care. Of risk factors examined, physical activity of less than 150 minutes per week was associated with lower odds of digital health care use, suggesting a potential role for telemonitoring to promote physical activity.^46^ Although evidence has been mixed on associations between race and ethnicity and telehealth use,^17,47,48^ our results show that Black or African American and Latino individuals had higher odds of using digital health care.^18,19,49^ English nonproficiency showed positive associations with digital health care use, consistent with prior work that has shown that Spanish speakers have higher telehealth use rates.^17^ These results may be on the account of outreach programs to traditionally underserved populations or a demonstration of digital health care capabilities in increasing health care access among underserved populations.^50^ Conversely, increased odds of digital health care use among traditionally underserved participants could reflect underlying disparities whereby they bear higher burden of disease and COVID-19 infections.^51,52,53^ Studies should replicate these findings using nationally representative samples and other data (eg, administrative claims).

County-level social vulnerability had both a direct and indirect association with digital health care use. Specifically, MHSVI was directly associated with telemonitoring use, whereas the direction and significance of associations between individual-level factors and digital health care use varied by MHSVI strata. While previous studies^17,19,45,49^ examined single place-based disadvantage metrics such as rurality or geographic divisions, we used the MHSVI, a multi-indicator assessment of county-level social vulnerability.^25^ To our knowledge, 2 studies used an earlier index, the Social Vulnerability Index, or its components to examine digital health care use.^54^ Both studies found that more vulnerable communities had higher use of audio-only visits to deliver care, but lower use of video-and-audio visits,^55^ and lower likelihood of adopting telemonitoring services.^22^ Previous studies^41,42,56,57^ suggested that health care organizations should dedicate resources to building interconnected infrastructures that are foundational to scaling up the provision of digital services. Research should unpack the associations between county-level factors and digital health care use. For instance, our results may reflect variations in access to or the quality of digital health care services available to residents of such counties.^58^ Differences in associations between individual-level factors and digital health care use by MHSVI strata are beneficial in identifying intervention targets for outreach and promotional activities to increase digital health care use. MHSVI is particularly useful because it encompasses social determinants of health (eg, socioeconomic status) and other vulnerabilities (eg, medical vulnerability) that are relevant to public health.^25^

Digital health literacy emerged as an important factor of digital health care use. In general, individuals with higher scores on digital health literacy domains had increased odds of digital health care use, including those residing in the most vulnerable counties. Exceptions included the negative association between the feeling-safe-and-in-control domain and digital health care use. One potential explanation is that people who are knowledgeable of security and privacy threats to personal health information have higher standards or perceive existing protections of their information as insufficient.^59^ Inconsistent with previous work,^17,18,19^ internet access was not associated with digital health care use, which could reflect the near-saturation of internet access and smart devices.^60,61^ Although previous studies^62^ noted persisting disparities among some underresourced populations, we found that disparities shifted from access to prerequisites of technology use including digital health literacy.^63,64^ Researchers should examine disparities in digital health literacy and its association with use of digital health care services. Policymakers should prioritize initiatives that strengthen digital health care infrastructure and digital health literacy to promote an accessible digital health care ecosystem.

### Strengths and Limitations

The study had strengths and limitations. We examined the interplay of individual-level and county-level indicators of disadvantage on use of 3 digital health care services that are often studied under the umbrella of telehealth. We used a comprehensive index of county-level social vulnerability rather than relying on a single metric.^65^ Nonprobability sampling limits generalizability of the results. We could not establish causality between factors examined and digital health care use in a cross-sectional study. Some factors had weak and/or inconsistent associations with digital health care use across imputed and complete case analyses. Responses to questions could be subject to misinterpretation and recall bias. Potential participants who lacked internet access might not have participated in the survey. The online survey platform calculates survey participation (rather than response) rate based on the number of targetable participants who qualify for and complete the survey and does not disclose information about nonresponders.

## Conclusions

While disparities in digital health care use were observed in association with traditional individual-level indicators of disadvantage, increased digital health care use among traditionally underserved participants found in this study is promising. Individual-level characteristics showed different associations with digital health care use based on county-level social vulnerability. Efforts are needed to ensure universal access to digital health care and to mitigate county-level vulnerability to future public health emergencies.

## Notes

Supplement 1. **eTable 1.** Target demographic composition of study sample based on NCHS population estimates **eTable 2.** Characteristics of excluded vs included participants in the analysis and those with missing vs complete data **eTable 3.** Multinomial logit model for use of digital health care services (N= 5444, imputed data) **eTable 4.** Associations between sociodemographic, health, and technology factors and telehealth use (n= 4318, complete case) **eTable 5.** Associations between sociodemographic, health, and technology factors and telemedicine use (n= 4318, complete case) **eTable 6.** Associations between sociodemographic, health, and technology factors and telemonitoring use (n= 4318, complete case)

Supplement 2.
Data Sharing Statement
