# Supplementary material for: Disparities in Digital Health Care Use in 2022
Source: JAMA Netw Open. 2025 Apr 17;8(4):e255359. doi: 10.1001/jamanetworkopen.2025.5359 (PMC12550827; doi:10.1001/jamanetworkopen.2025.5359)
Supplement: Supplement 1. — eTable 1. Target demographic composition of study sample based on NCHS population estimates eTable 2. Characteristics of excluded vs included participants in the analysis and those with missing vs complete data eTable 3. Multinomial logit model for use of digital health care services (N= 5444, imputed data) eTable 4. Associations between sociodemographic, health, and technology factors and telehealth use (n= 4318, complete case) eTable 5. Associations between sociodemographic, health, and technology factors and telemedicine use (n= 4318, complete case) eTable 6. Associations between sociodemographic, health, and technology factors and telemonitoring use (n= 4318, complete case) [file jamanetwopen-e255359-s001.pdf]

Supplemental Online Content

Wakeman M, Buckman DW, El-Toukhy S. Disparities in digital health care use in 2022. *JAMA Netw Open.* 2025;8(4):e255359. doi:10.1001/jamanetworkopen.2025.5359

eTable 1. Target demographic composition of study sample based on NCHS population estimates

eTable 2. Characteristics of excluded vs included participants in the analysis and those with missing vs complete data

eTable 3. Multinomial logit model for use of digital health care services (N= 5444, imputed data)

eTable 4. Associations between sociodemographic, health, and technology factors and telehealth use (n= 4318, complete case)

eTable 5. Associations between sociodemographic, health, and technology factors and telemedicine use (n= 4318, complete case)

eTable 6. Associations between sociodemographic, health, and technology factors and telemonitoring use (n= 4318, complete case)

This supplemental material has been provided by the authors to give readers additional information about their work.

© 2025 Wakeman M et al. *JAMA Network Open*.

eTable 1. Target demographic composition of study sample based on NCHS population estimates.

		Most-vulnerable counties (MHSVI top quartile)	Least-vulnerable counties (MHSVI bottom quartile)
		**Sample *n*= 2750 (%)**	**Sample *n*= 2750 (%)**
**Sex**	Female	1410 (51.26)	1406 (51.12)
	Male	1340 (48.74)	1344 (48.88)
**Age**	18-29	620 (22.56)	533 (19.40)
	30-44	677 (24.61)	662 (24.06)
	45-59	670 (24.36)	724 (26.32)
	60+	783 (28.46)	831 (30.22)
**Race and Ethnicity**	NH AIAN	50 (1.82)	12 (0.45)
	NH Asian or PI	80 (2.89)	146 (5.31)
	NH Black or AA	577 (20.98)	180 (6.53)
	Hispanic or Latino	570 (20.73)	196 (7.14)
	NH White	1473 (53.58)	2216 (80.57)

NCHS= National Center for Health Statistics; MHSVI= Minority Health Social Vulnerability Index; NH= Non-Hispanic; AIAN= American Indian, Alaska Native; PI = Pacific Islander; AA= African American.

© 2025 Wakeman M et al. *JAMA Network Open*.

eTable 2. Characteristics of excluded vs included participants in the analysis and those with missing vs complete data

	Excluded (*n*= 115)[a]	Included (*n*= 5444)[a]	*p*	With missing data (*n*= 1126)[b]	With complete data (*n*= 4318)[b]	*p*
	**No. (%)**	**No. (%)**		**No. (%)**	**No. (%)**	
**Age[c]**			NA			**<0.0001**
18-29	NA	1222 (22.45)		415 (36.86)	807 (18.69)	
30-44	NA	1517 (27.87)		308 (27.35)	1209 (28.00)	
45-59	NA	1387 (25.48)		233 (20.69)	1154 (26.73)	
≥60	NA	1318 (24.21)		170 (15.10)	1148 (26.59)	
**Sex**						**<0.0001**
Female	57 (49.57)	2927 (53.77)		714 (63.41)	2213 (51.25)	
Male	58 (50.43)	2517 (46.23)		412 (36.59)	2105 (48.75)	
**Sexual orientation**			**0.001**			**<0.0001**
Heterosexual	90 (78.26)	4746 (87.18)		860 (85.57)	3886 (90.00)	
Non-heterosexual	24 (20.87)	577 (10.60)		145 (14.43)	432 (10.00)	
Prefer not to answer / I don't know	1 (0.87)	121 (2.22)				
**Race and ethnicity**			0.33			**<0.0001**
NH AIAN	0 (0)	58 (1.07)		9 (0.80)	49 (1.13)	
NH Asian or NHPI	2 (1.74)	208 (3.82)		57 (5.06)	151 (3.50)	
NH Black or AA	14 (12.17)	798 (14.66)		228 (20.25)	570 (13.20)	
Hispanic or Latino	23 (20.00)	838 (15.39)		233 (20.69)	605 (14.01)	
NH White	76 (66.09)	3542 (65.06)		599 (53.20)	2943 (68.16)	
**Education**			0.60			**0.004**
<High school	11 (9.57)	279 (5.12)		97 (8.96)	182 (4.21)	
High school graduate	39 (33.91)	1444 (26.52)		366 (33.80)	1078 (24.97)	
Vocational school, some college	34 (29.57)	1978 (36.33)		367 (33.89)	1611 (37.31)	
College graduate or higher	28 (24.35)	1700 (31.23)		253 (23.36)	1447 (33.51)	
Prefer not to answer / I don't know	3 (2.61)	43 (0.79)		NA	NA	
**Income (in 2021)**			0.83			0.71
<$20,000	27 (23.48)	1215 (22.32)		294 (38.74)	921 (21.33)	
$20,000 to $49,999	34 (29.57)	1588 (29.17)		233 (30.70)	1355 (31.38)	
$50,000 to $74,999	20 (17.39)	891 (16.37)		102 (13.44)	789 (18.27)	
≥$75,000	27 (23.48)	1383 (25.40)		130 (17.13)	1253 (29.02)	
Prefer not to answer / I don't know	7 (6.09)	367 (6.74)				
**English proficiency**			**<0.0001**			**<0.0001**

© 2025 Wakeman M et al. *JAMA Network Open.*

	Excluded (*n*= 115)[a]	Included (*n*= 5444)[a]	*p*	With missing data (*n*= 1126)[b]	With complete data (*n*= 4318)[b]	*p*
	**No. (%)**	**No. (%)**		**No. (%)**	**No. (%)**	
Very well	92 (80.00)	4984 (91.55)		930 (82.59)	4054 (93.89)	
Well, not well, not at all	23 (20.00)	460 (8.45)		196 (17.41)	264 (6.11)	
**Health insurance**			0.92			**<0.0001**
Insured	102 (88.70)	4844 (88.98)		938 (83.30)	3906 (90.46)	
Uninsured	13 (11.30)	600 (11.02)		188 (16.70)	412 (9.54)	
**General health**			0.32			0.13
Excellent, very good, good	83 (72.17)	4144 (76.12)		838 (74.42)	3306 (76.56)	
Fair, poor	32 (27.83)	1300 (23.88)		288 (25.58)	1012 (23.44)	
**Mental health**			0.15			0.16
Excellent, very good, good	77 (66.96)	3972 (72.96)		803 (71.31)	3169 (73.39)	
Fair, poor	38 (33.04)	1472 (27.04)		323 (28.69)	1149 (26.61)	
**Presence of underlying medical conditions**			0.71			**<0.0001**
Yes	38 (33.04)	1887 (34.66)		282 (25.04)	1605 (37.17)	
No	77 (66.96)	3557 (65.34)		844 (74.96)	2713 (62.83)	
**Ever tested positive for COVID-19**			0.08			**0.007**
Yes	40 (34.78)	1396 (25.64)		227 (22.95)	1169 (27.07)	
No	72 (62.61)	3911 (71.84)		762 (77.05)	3149 (72.93)	
Prefer not to answer / I don't know	3 (2.61)	137 (2.52)		NA	NA	
**Past year in-person visit(s) with healthcare clinician**			0.06			**<0.0001**
0 times	16 (13.91)	1146 (21.05)		302 (26.82)	844 (19.55)	
1+ times	99 (86.09)	4298 (78.95)		824 (73.18)	3474 (80.45)	
**Have a primary care clinician**			0.36			**<0.0001**
Yes	60 (52.17)	2609 (47.92)		421 (37.39)	2188 (50.67)	
No	55 (47.83)	2835 (52.08)		705 (62.61)	2130 (49.33)	
**BMI[d]**			0.06			0.09
Healthy	37 (32.17)	1385 (25.44)		131 (33.08)	1254 (29.04)	
Unhealthy	58 (50.43)	3329 (61.15)		265 (66.92)	3064 (70.96)	
Unknown	20 (17.39)	730 (13.41)		NA	NA	
**Physical activity**			**0.04**			**0.002**
Sufficient	84 (73.04)	3494 (64.18)		680 (60.39)	2814 (65.17)	
Insufficient	31 (26.96)	1950 (35.82)		446 (39.61)	1504 (34.83)	

© 2025 Wakeman M et al. *JAMA Network Open.*

	Excluded (*n*= 115)[a]	Included (*n*= 5444)[a]	*p*	With missing data (*n*= 1126)[b]	With complete data (*n*= 4318)[b]	*p*
	**No. (%)**	**No. (%)**		**No. (%)**	**No. (%)**	
**Past month cigarette smoking**			**<0.0001**			0.07
Yes	60 (52.17)	1601 (29.41)		355 (31.53)	1246 (28.86)	
No	55 (47.83)	3843 (70.59)		771 (68.47)	3072 (71.14)	
**Past month e-cigarette use**			**<0.0001**			**<0.0001**
Yes	45 (39.13)	918 (16.86)		265 (23.53)	653 (15.12)	
No	70 (60.87)	4526 (83.14)		861 (76.47)	3665 (84.88)	
**Alcohol misuse[c]**			NA			**<0.0001**
Yes	NA	350 (6.43)		58 (5.15)	292 (6.76)	
No	NA	4848 (89.05)		958 (85.08)	3890 (90.09)	
Not applicable (<21 years)	NA	246 (4.52)		110 (9.77)	136 (3.15)	
**Past month marijuana or cannabis use**			**<0.0001**			**0.0004**
Yes	52 (45.22)	1350 (24.80)		325 (28.86)	1025 (23.74)	
No	63 (54.78)	4094 (75.20)		801 (71.14)	3293 (76.26)	
**Have internet access**			0.33			**<0.0001**
Yes	114 (99.13)	5325 (97.81)		1069 (94.94)	4256 (98.56)	
No	1 (0.87)	119 (2.19)		57 (5.06)	62 (1.44)	
**Digital health literacy[e]**						
Using technology to process health information	2.944 (0.055)	2.911 (0.007)	0.53	2.826 (0.017)	2.934 (0.008)	**<0.0001**
Understanding of health concepts and language	2.967 (0.051)	2.989 (0.006)	0.66	2.879 (0.015)	3.018 (0.007)	**<0.0001**
Ability to actively engage with digital services	2.972 (0.052)	2.954 (0.007)	0.74	2.861 (0.017)	2.979 (0.008)	**<0.0001**
Feel safe and in control	2.871 (0.052)	2.830 (0.008)	0.47	2.759 (0.018)	2.849 (0.009)	**<0.0001**
Motivated to engage with digital services	2.932 (0.053)	2.887 (0.007)	0.40	2.786 (0.017)	2.914 (0.008)	**<0.0001**
Access to digital services that work	2.963 (0.047)	2.928 (0.006)	0.46	2.831 (0.015)	2.953 (0.007)	**<0.0001**
Digital services that suit individual needs	2.863 (0.054)	2.804 (0.008)	0.30	2.745 (0.018)	2.820 (0.009)	**0.0003**

NH= Non-Hispanic, AIAN= American Indian or Alaska Native, NHPI= Native Hawaiian or Pacific Islander, AA= African American, BMI = Body Mass Index.
Bolded cells represent significant results.

© 2025 Wakeman M et al. *JAMA Network Open.*

Healthy BMI ranged from 18.5 to <25 kg/m^2, whereas unhealthy BMI was either <18.5 or ≥25 kg/m2; sufficient physical activity was ≥150 minutes per week, whereas insufficient physical activity was <150 minutes per week; cigarette smoking, e-cigarette use, and marijuana use were coded “yes” when participants self-reported use on ≥1 days in the past month; alcohol misuse was defined as ≥3 drinks/day for men and ≥2 drinks/day for women, otherwise alcohol misuse was coded no.

[a] Based on a sample of 5559 participants.
[b] Based on a sample of 5444 participants.
[c] Excluded participants inconsistently reported their age. Frequencies, percentages, and chi-square were not produced.
[d] BMI was derived from height and weight with 0.25% exclusive missingness on height (*n*= 14) and 13.15% missingness on weight (*n*= 716).
[e] Cells include means and standard errors. Digital health literacy was measured using a 35-item, 1 to 4-point scale with higher scores indicating higher digital health literacy. Domain scores were averaged across items under each domain

© 2025 Wakeman M et al. *JAMA Network Open.*

eTable 3. Multinomial logit model for use of digital healthcare services (*N*= 5444, imputed data).

Characteristics	Telehealth only (*n*= 1232)	Telemedicine only (*n*= 179)	Telemonitoring only (*n*= 115)	Any two Services (*n*= 986)	All three Services (*n*= 573)
	**aOR (95% CI)**	**aOR (95% CI)**	**aOR (95% CI)**	**aOR (95% CI)**	**aOR (95% CI)**
**Age**	1.00 (0.99, 1.01)	**0.98 (0.97, 0.99)**	0.99 (0.97, 1.00)	**0.98 (0.97, 0.99)**	**0.97 (0.97, 0.98)**
**Sex**					
Female	1.12 (0.96, 1.32)	1.06 (0.77, 1.46)	0.92 (0.61, 1.37)	1.08 (0.90, 1.28)	0.97 (0.77, 1.22)
Male	Ref	Ref	Ref	Ref	Ref
**Sexual orientation**					
Heterosexual	Ref	Ref	Ref	Ref	Ref
Non-heterosexual	1.06 (0.81, 1.38)	1.24 (0.78, 1.96)	0.70 (0.31, 1.57)	**1.43 (1.10, 1.85)**	1.33 (0.95, 1.87)
**Race and ethnicity**					
NH Black or AA	**1.35 (1.07, 1.72)**	1.55 (0.98, 2.45)	1.48 (0.87, 2.51)	**1.47 (1.14, 1.89)**	**2.09 (1.51, 2.89)**
Hispanic or Latino	**1.54 (1.22, 1.95)**	1.59 (1.00, 2.52)	1.17 (0.63, 2.19)	1.20 (0.93, 1.55)	**2.22 (1.64, 3.01)**
NH Other[a]	**1.52 (1.10, 2.10)**	0.83 (0.34, 2.01)	1.68 (0.75, 3.74)	1.08 (0.73, 1.61)	1.41 (0.87, 2.30)
NH White	Ref	Ref	Ref	Ref	Ref
**Education**	1.07 (0.97, 1.18)	**1.33 (1.06, 1.66)**	0.97 (0.74, 1.27)	**1.16 (1.04, 1.29)**	1.10 (0.95, 1.27)
**Income (in 2021)**	1.01 (0.93, 1.09)	0.98 (0.82, 1.17)	1.12 (0.91, 1.37)	0.92 (0.84, 1.01)	1.03 (0.91, 1.16)
**English proficiency**					
Very well	Ref	Ref	Ref	Ref	Ref
Well, not well, not at all	0.98 (0.71, 1.33)	0.67 (0.32, 1.41)	0.96 (0.43, 2.12)	**1.81 (1.36, 2.42)**	**2.16 (1.53, 3.04)**
**Health insurance**					
Insured	Ref	Ref	Ref	Ref	Ref
Uninsured	**0.51 (0.38, 0.67)**	0.68 (0.38, 1.19)	0.92 (0.48, 1.75)	**0.73 (0.55, 0.96)**	**0.64 (0.44, 0.94)**
**General health**					
Excellent, very good, good	Ref	Ref	Ref	Ref	Ref

© 2025 Wakeman M et al. *JAMA Network Open.*

Characteristics	Telehealth only (*n*= 1232)	Telemedicine only (*n*= 179)	Telemonitoring only (*n*= 115)	Any two Services (*n*= 986)	All three Services (*n*= 573)
	**aOR (95% CI)**	**aOR (95% CI)**	**aOR (95% CI)**	**aOR (95% CI)**	**aOR (95% CI)**
Fair, poor	**1.32 (1.08, 1.61)**	0.73 (0.45, 1.16)	**2.18 (1.36, 3.50)**	**1.44 (1.16, 1.79)**	1.31 (0.96, 1.79)
**Mental health**					
Excellent, very good, good	Ref	Ref	Ref	Ref	Ref
Fair, poor	**1.32 (1.09, 1.60)**	**2.04 (1.40, 2.97)**	0.81 (0.50, 1.31)	**1.26 (1.02, 1.55)**	0.83 (0.61, 1.11)
**Having a primary care clincian**					
Yes	Ref	Ref	Ref	Ref	Ref
No	**0.79 (0.66, 0.93)**	**0.64 (0.45, 0.90)**	0.76 (0.47, 1.22)	**0.61 (0.51, 0.74)**	**0.41 (0.32, 0.52)**
**Presence of underlying medical conditions**					
Yes	1.13 (0.95, 1.35)	0.91 (0.63, 1.31)	0.99 (0.60, 1.64)	1.12 (0.92, 1.37)	0.87 (0.67, 1.13)
No	Ref	Ref	Ref	Ref	Ref
**Past year in-person visit(s) with health care clinician**					
0 times	Ref	Ref	Ref	Ref	Ref
1+ times	**3.76 (3.02, 4.67)**	**2.34 (1.53, 3.59)**	1.56 (0.97, 2.51)	**5.99 (4.53, 7.92)**	**19.58 (10.79, 35.53)**
**Ever tested positive for COVID-19**					
Yes	**1.56 (1.32, 1.84)**	0.82 (0.56, 1.21)	**1.72 (1.13, 2.61)**	**1.35 (1.12, 1.63)**	**1.39 (1.08, 1.79)**
No	Ref	Ref	Ref	Ref	Ref
**BMI**					
Healthy	Ref	Ref	Ref	Ref	Ref
Unhealthy	1.07 (0.89, 1.27)	1.18 (0.82, 1.69)	1.10 (0.68, 1.80)	**1.28 (1.05, 1.55)**	1.18 (0.90, 1.54)
**Physical activity**					
Sufficient	Ref	Ref	Ref	Ref	Ref
Insufficient	0.91 (0.78, 1.07)	0.76 (0.54, 1.07)	0.77 (0.51, 1.15)	**0.67 (0.56, 0.81)**	**0.45 (0.34, 0.61)**
**Past month cigarette smoking**					
Yes	**1.32 (1.07, 1.62)**	1.29 (0.87, 1.92)	0.98 (0.57, 1.69)	**1.68 (1.36, 2.09)**	**2.63 (1.99, 3.47)**
No	Ref	Ref	Ref	Ref	Ref

© 2025 Wakeman M et al. *JAMA Network Open.*

Characteristics	Telehealth only (*n*= 1232)	Telemedicine only (*n*= 179)	Telemonitoring only (*n*= 115)	Any two Services (*n*= 986)	All three Services (*n*= 573)
	aOR (95% CI)	aOR (95% CI)	aOR (95% CI)	aOR (95% CI)	aOR (95% CI)
**Past month e-cigarette use**					
Yes	**1.49 (1.13, 1.97)**	1.01 (0.61, 1.67)	1.66 (0.90, 3.07)	**1.90 (1.46, 2.48)**	**3.93 (2.92, 5.30)**
No	Ref	Ref	Ref	Ref	Ref
**Alcohol misuse**					
Yes	0.81 (0.59, 1.12)	0.62 (0.30, 1.30)	0.53 (0.19, 1.48)	0.87 (0.62, 1.22)	0.92 (0.59, 1.42)
No	Ref	Ref	Ref	Ref	Ref
Not applicable (<21 years)	0.87 (0.56, 1.37)	1.09 (0.53, 2.26)	1.11 (0.44, 2.84)	1.15 (0.78, 1.70)	1.02 (0.62, 1.69)
**Past month marijuana or cannabis use**					
Yes	1.13 (0.90, 1.40)	**1.75 (1.16, 2.64)**	1.23 (0.74, 2.02)	**1.48 (1.19, 1.84)**	**2.31 (1.77, 3.02)**
No	Ref	Ref	Ref	Ref	Ref
**Have internet access**					
Yes	Ref	Ref	Ref	Ref	Ref
No	1.18 (0.66, 2.10)	0.68 (0.16, 2.85)	1.00 (0.23, 4.38)	1.41 (0.80, 2.47)	1.21 (0.55, 2.66)
**Digital health literacy**					
Using technology to process health information	1.17 (0.89, 1.54)	1.60 (0.91, 2.79)	1.27 (0.61, 2.64)	**1.97 (1.45, 2.68)**	**1.56 (1.02, 2.39)**
Understanding of health concepts and language	1.06 (0.82, 1.38)	0.61 (0.35, 1.07)	0.92 (0.45, 1.86)	0.92 (0.68, 1.23)	0.82 (0.56, 1.21)
Ability to actively engage with digital services	0.90 (0.72, 1.14)	0.78 (0.48, 1.24)	0.59 (0.35, 1.00)	**0.69 (0.54, 0.89)**	**0.57 (0.40, 0.81)**
Feel safe and in control	**0.83 (0.69, 0.99)**	1.04 (0.69, 1.56)	1.31 (0.80, 2.13)	0.88 (0.72, 1.07)	1.09 (0.82, 1.43)
Motivated to engage with digital services	1.04 (0.78, 1.37)	1.28 (0.68, 2.39)	0.80 (0.36, 1.74)	**1.45 (1.07, 1.96)**	**1.97 (1.28, 3.03)**
Access to digital services that work	1.23 (0.94, 1.62)	0.74 (0.39, 1.38)	1.25 (0.57, 2.72)	0.94 (0.69, 1.28)	0.81 (0.53, 1.25)
Digital services that suit individual needs	1.29 (1.02, 1.64)	1.59 (0.98, 2.59)	**1.86 (1.03, 3.37)**	**1.36 (1.05, 1.75)**	**2.28 (1.61, 3.24)**
**MHSVI**					

© 2025 Wakeman M et al. *JAMA Network Open.*

Characteristics	Telehealth only (*n*= 1232)	Telemedicine only (*n*= 179)	Telemonitoring only (*n*= 115)	Any two Services (*n*= 986)	All three Services (*n*= 573)
	**aOR (95% CI)**	**aOR (95% CI)**	**aOR (95% CI)**	**aOR (95% CI)**	**aOR (95% CI)**
Most-vulnerable counties	1.00 (0.85, 1.17)	0.89 (0.62, 1.27)	1.40 (0.93, 2.09)	1.08 (0.90, 1.30)	**1.39 (1.09, 1.78)**
Least-vulnerable counties	Ref	Ref	Ref	Ref	Ref

aOR= adjusted odds ratio, CI= confidence interval, NH= Non-Hispanic, AA = African American, BMI = Body Mass Index, MHSVI= Minority Health Social Vulnerability Index, Ref= reference group.
Most-vulnerable counties fall in the top quartile of the MHSVI, whereas least-vulnerable counties fall in the bottom quartile of MHSVI.
Bolded cells represent significant results.
Reference group for multinomial logit model was those who did not use any digital healthcare service (*n*= 2359).
Income and education were entered as ordinal variables; age and digital literacy were entered as continuous variables; all other variables (e.g., race/ethnicity, sex) were entered as categorical variables. Healthy BMI ranged from 18.5 to <25 kg/m^2, whereas unhealthy BMI was either <18.5 or ≥25 kg/m2; sufficient physical activity was ≥150 minutes per week, whereas insufficient physical activity was <150 minutes per week; cigarette smoking, e-cigarette use, and marijuana use were coded “yes” when participants self-reported use on ≥1 days in the past month; alcohol misuse was defined as ≥3 drinks/day for men and ≥2 drinks/day for women, otherwise alcohol misuse was coded no.
[a] Non-Hispanic Asian, Native Hawaiian, Pacific Islander, American Indian, and Alaska Native individuals were collapsed into NH Other.

eTable 4. Associations between sociodemographic, health, and technology factors and telehealth use (*n*= 4318, complete case).

	Overall (*N*= 4318)		MHSVI most-vulnerable counties (*n*= 2199)		MHSVI least-vulnerable counties (*n*= 2119)	
	PM (95% CI)	aOR (95% CI)	PM (95% CI)	aOR (95% CI)	PM (95% CI)	aOR (95% CI)
**Age**						
18	0.52 (0.49, 0.56)	0.99 (0.99, 1.00)	0.59 (0.54, 0.63)	**0.98 (0.98, 0.99)**	0.46 (0.41, 0.51)	1.00 (0.99, 1.00)
30	0.51 (0.49, 0.54)		0.56 (0.53, 0.59)		0.46 (0.43, 0.50)	
45	0.50 (0.48, 0.51)		0.53 (0.51, 0.55)		0.47 (0.44, 0.49)	
60	0.49 (0.47, 0.51)		0.49 (0.46, 0.52)		0.47 (0.44, 0.49)	
**Sex**						
Female	0.51 (0.49, 0.53)	1.10 (0.95, 1.26)	0.54 (0.51, 0.57)	1.15 (0.94, 1.42)	0.47 (0.44, 0.50)	1.04 (0.85, 1.27)
Male	0.49 (0.46, 0.51)	Ref	0.51 (0.48, 0.54)	Ref	0.46 (0.43, 0.49)	Ref
**Sexual orientation**						
Heterosexual	0.49 (0.48, 0.51)	Ref	0.53 (0.51, 0.55)	Ref	0.46 (0.44, 0.48)	Ref
Non-heterosexual	0.51 (0.46, 0.55)	1.07 (0.85, 1.34)	0.50 (0.45, 0.56)	0.89 (0.65, 1.22)	0.52 (0.45, 0.59)	1.32 (0.94, 1.85)
**Race and ethnicity**						
NH Black or AA	0.54 (0.50, 0.58)	**1.34 (1.08, 1.66)**	0.54 (0.50, 0.58)	1.22 (0.94, 1.58)	0.61 (0.52, 0.69)	**2.08 (1.33, 3.23)**
Hispanic or Latino	0.54 (0.50, 0.58)	**1.35 (1.10, 1.67)**	0.56 (0.51, 0.60)	**1.34 (1.02, 1.76)**	0.48 (0.41, 0.56)	1.16 (0.80, 1.67)
NH Other[a]	0.56 (0.50, 0.63)	**1.53 (1.11, 2.12)**	0.57 (0.48, 0.66)	1.43 (0.87, 2.34)	0.55 (0.46, 0.63)	**1.56 (1.01, 2.41)**
NH White	0.48 (0.46, 0.49)	Ref	0.50 (0.47, 0.53)	Ref	0.45 (0.43, 0.48)	Ref
**Education**						
<High school	0.47 (0.43, 0.51)	1.07 (0.98, 1.17)	0.45 (0.40, 0.50)	**1.22 (1.08, 1.38)**	0.50 (0.43, 0.56)	0.93 (0.82, 1.07)
High school graduate	0.48 (0.46, 0.50)		0.49 (0.46, 0.52)		0.48 (0.44, 0.52)	

© 2025 Wakeman M et al. *JAMA Network Open.*

	Overall (*N*= 4318)		MHSVI most-vulnerable counties (*n*= 2199)		MHSVI least-vulnerable counties (*n*= 2119)	
	PM (95% CI)	aOR (95% CI)	PM (95% CI)	aOR (95% CI)	PM (95% CI)	aOR (95% CI)
Vocational school, some college	0.50 (0.48, 0.51)		0.53 (0.51, 0.55)		0.47 (0.45, 0.49)	
College graduate or higher	0.51 (0.49, 0.53)		0.57 (0.54, 0.60)		0.46 (0.43, 0.49)	
**Income (in 2021)**						
<$20,000	0.50 (0.47, 0.53)	0.98 (0.91, 1.05)	0.51 (0.48, 0.55)	1.04 (0.93, 1.15)	0.49 (0.45, 0.54)	0.94 (0.85, 1.04)
$20,000 to $49,999	0.50 (0.48, 0.52)		0.52 (0.50, 0.54)		0.48 (0.45, 0.51)	
$50,000 to $74,999	0.50 (0.48, 0.51)		0.53 (0.50, 0.56)		0.47 (0.44, 0.49)	
≥$75,000	0.49 (0.47, 0.52)		0.54 (0.50, 0.58)		0.45 (0.42, 0.48)	
**English proficiency**						
Very well	0.49 (0.48, 0.51)	Ref	0.52 (0.50, 0.54)	Ref	0.47 (0.44, 0.49)	Ref
Well, not well, not at all	0.53 (0.47, 0.58)	1.15 (0.87, 1.53)	0.56 (0.48, 0.63)	1.19 (0.81, 1.73)	0.49 (0.40, 0.58)	1.13 (0.73, 1.75)
**Health insurance**						
Insured	0.50 (0.49, 0.52)	Ref	0.53 (0.51, 0.55)	Ref	0.47 (0.45, 0.49)	Ref
Uninsured	0.42 (0.37, 0.47)	**0.66 (0.52, 0.84)**	0.48 (0.42, 0.53)	0.76 (0.56, 1.01)	0.35 (0.26, 0.44)	**0.54 (0.34, 0.85)**
**General health**						
Excellent, very good, good	0.48 (0.46, 0.50)	Ref	0.51 (0.48, 0.53)	Ref	0.45 (0.43, 0.48)	Ref
Fair, poor	0.54 (0.51, 0.58)	**1.34 (1.12, 1.61)**	0.58 (0.54, 0.62)	**1.44 (1.11, 1.85)**	0.51 (0.46, 0.56)	**1.32 (1.01, 1.72)**
**Mental health**						
Excellent, very good, good	0.49 (0.47, 0.51)	Ref	0.52 (0.50, 0.55)	Ref	0.45 (0.43, 0.48)	Ref
Fair, poor	0.52 (0.49, 0.55)	1.16 (0.98, 1.38)	0.53 (0.49, 0.57)	1.01 (0.79, 1.29)	0.52 (0.47, 0.56)	**1.35 (1.05, 1.73)**
**Having a primary care clinician**						

© 2025 Wakeman M et al. *JAMA Network Open.*

	Overall (*N*= 4318)		MHSVI most-vulnerable counties (*n*= 2199)		MHSVI least-vulnerable counties (*n*= 2119)	
	PM (95% CI)	aOR (95% CI)	PM (95% CI)	aOR (95% CI)	PM (95% CI)	aOR (95% CI)
Yes	0.53 (0.51, 0.55)	Ref	0.58 (0.55, 0.61)	Ref	0.49 (0.46, 0.52)	Ref
No	0.45 (0.43, 0.48)	**0.68 (0.59, 0.79)**	0.47 (0.44, 0.50)	**0.58 (0.47, 0.72)**	0.44 (0.40, 0.47)	**0.76 (0.62, 0.94)**
**Presence of underlying medical conditions**						
Yes	0.51 (0.48, 0.53)	1.08 (0.92, 1.27)	0.51 (0.48, 0.55)	0.91 (0.72, 1.15)	0.50 (0.46, 0.53)	1.26 (1.00, 1.57)
No	0.49 (0.47, 0.51)	Ref	0.53 (0.50, 0.56)	Ref	0.45 (0.42, 0.48)	Ref
**Past year in-person visit(s) with health care clinician**						
0 times	0.25 (0.22, 0.29)	Ref	0.24 (0.20, 0.29)	Ref	0.27 (0.22, 0.32)	Ref
1+ times	0.55 (0.53, 0.57)	**4.16 (3.41, 5.08)**	0.59 (0.57, 0.61)	**5.55 (4.18, 7.36)**	0.51 (0.49, 0.53)	**3.17 (2.38, 4.22)**
**Ever tested positive for COVID-19**						
Yes	0.55 (0.52, 0.57)	**1.38 (1.19, 1.60)**	0.58 (0.54, 0.61)	**1.45 (1.17, 1.79)**	0.52 (0.48, 0.56)	**1.39 (1.12, 1.72)**
No	0.48 (0.46, 0.50)	Ref	0.50 (0.48, 0.53)	Ref	0.45 (0.42, 0.47)	Ref
**BMI**						
Healthy	0.48 (0.45, 0.50)	Ref	0.48 (0.44, 0.52)	Ref	0.48 (0.44, 0.52)	Ref
Unhealthy	0.50 (0.49, 0.52)	1.13 (0.97, 1.32)	0.54 (0.52, 0.57)	**1.36 (1.09, 1.70)**	0.46 (0.44, 0.49)	0.91 (0.74, 1.13)
**Physical activity**						
Sufficient	0.51 (0.49, 0.53)	Ref	0.55 (0.52, 0.57)	Ref	0.47 (0.44, 0.49)	Ref
Insufficient	0.48 (0.45, 0.50)	**0.85 (0.73, 0.98)**	0.48 (0.45, 0.52)	**0.72 (0.58, 0.89)**	0.47 (0.43, 0.50)	1.01 (0.82, 1.24)
**Past month cigarette smoking**						
Yes	0.56 (0.53, 0.59)	**1.49 (1.24, 1.78)**	0.59 (0.55, 0.62)	**1.64 (1.28, 2.09)**	0.52 (0.47, 0.58)	**1.39 (1.06, 1.84)**

	Overall (*N*= 4318)		MHSVI most-vulnerable counties (*n*= 2199)		MHSVI least-vulnerable counties (*n*= 2119)	
	PM (95% CI)	aOR (95% CI)	PM (95% CI)	aOR (95% CI)	PM (95% CI)	aOR (95% CI)
No	0.47 (0.46, 0.49)	Ref	0.49 (0.46, 0.52)	Ref	0.45 (0.43, 0.48)	Ref
**Past month e-cigarette use**						
Yes	0.60 (0.56, 0.64)	**1.80 (1.43, 2.26)**	0.62 (0.56, 0.67)	**1.74 (1.28, 2.38)**	0.57 (0.50, 0.64)	**1.74 (1.22, 2.49)**
No	0.48 (0.46, 0.50)	Ref	0.51 (0.48, 0.53)	Ref	0.45 (0.43, 0.48)	Ref
**Alcohol misuse**						
Yes	0.47 (0.42, 0.53)	0.88 (0.67, 1.15)	0.50 (0.42, 0.58)	0.87 (0.59, 1.30)	0.43 (0.36, 0.51)	0.82 (0.56, 1.20)
No	0.50 (0.48, 0.51)	Ref	0.52 (0.50, 0.55)	Ref	0.47 (0.45, 0.49)	Ref
Not applicable (<21 years)	0.49 (0.41, 0.57)	0.93 (0.63, 1.39)	0.57 (0.46, 0.67)	1.24 (0.69, 2.23)	0.39 (0.28, 0.51)	0.67 (0.38, 1.19)
**Past month marijuana or cannabis use**						
Yes	0.54 (0.50, 0.57)	**1.27 (1.06, 1.52)**	0.57 (0.52, 0.61)	**1.32 (1.02, 1.70)**	0.50 (0.45, 0.55)	1.20 (0.91, 1.57)
No	0.49 (0.47, 0.50)	Ref	0.51 (0.49, 0.54)	Ref	0.46 (0.44, 0.48)	Ref
**Have internet access**						
Yes	0.50 (0.48, 0.51)	Ref	0.52 (0.50, 0.54)	Ref	0.47 (0.45, 0.49)	Ref
No	0.54 (0.41, 0.65)	1.21 (0.67, 2.21)	0.60 (0.46, 0.73)	1.51 (0.70, 3.26)	0.40 (0.22, 0.61)	0.72 (0.26, 1.99)
**Digital health literacy**						
Using technology to process health information	0.48 (0.46, 0.50)<br>0.51 (0.49, 0.53)	1.27 (0.98, 1.63)	0.51 (0.48, 0.54)<br>0.53 (0.51, 0.56)	1.21 (0.84, 1.73)	0.45 (0.42, 0.48)<br>0.48 (0.45, 0.51)	1.25 (0.87, 1.79)
Understanding of health concepts and language	0.49 (0.47, 0.51)<br>0.50 (0.48, 0.52)	1.07 (0.84, 1.35)	0.53 (0.50, 0.55)<br>0.52 (0.50, 0.55)	0.91 (0.64, 1.28)	0.45 (0.43, 0.48)<br>0.48 (0.45, 0.51)	1.37 (0.97, 1.92)
Ability to actively engage with digital services	0.51 (0.49, 0.53)	0.82 (0.67, 1.01)	0.54 (0.51, 0.57)	0.85 (0.63, 1.15)	0.48 (0.45, 0.51)	0.80 (0.60, 1.08)

© 2025 Wakeman M et al. *JAMA Network Open.*

	Overall (*N*= 4318)		MHSVI most-vulnerable counties (*n*= 2199)		MHSVI least-vulnerable counties (*n*= 2119)	
	**PM (95% CI)**	**aOR (95% CI)**	**PM (95% CI)**	**aOR (95% CI)**	**PM (95% CI)**	**aOR (95% CI)**
	0.49 (0.47, 0.51)		0.52 (0.50, 0.54)		0.46 (0.43, 0.48)	
Feel safe and in control	0.50 (0.49, 0.52)	0.89 (0.76, 1.04)	0.54 (0.52, 0.57)	**0.72 (0.57, 0.93)**	0.46 (0.44, 0.49)	1.04 (0.84, 1.30)
	0.49 (0.47, 0.51)		0.51 (0.48, 0.53)		0.47 (0.44, 0.50)	
Motivated to engage with digital services	0.48 (0.45, 0.50)	**1.35 (1.04, 1.74)**	0.50 (0.47, 0.53)	1.43 (0.98, 2.09)	0.45 (0.42, 0.48)	1.30 (0.91, 1.85)
	0.51 (0.49, 0.54)		0.54 (0.51, 0.57)		0.48 (0.45, 0.52)	
Access to digital services that work	0.49 (0.47, 0.51)	1.08 (0.84, 1.40)	0.52 (0.48, 0.55)	1.16 (0.79, 1.69)	0.47 (0.44, 0.50)	1.03 (0.72, 1.46)
	0.50 (0.48, 0.52)		0.53 (0.50, 0.56)		0.47 (0.44, 0.50)	
Digital services that suit individual needs	0.48 (0.46, 0.50)	**1.26 (1.02, 1.56)**	0.50 (0.47, 0.53)	1.35 (0.99, 1.83)	0.45 (0.43, 0.48)	1.23 (0.91, 1.68)
	0.51 (0.49, 0.52)		0.53 (0.51, 0.56)		0.48 (0.45, 0.50)	
**MHSVI**						
Most-vulnerable counties	0.50 (0.48, 0.52)	1.03 (0.89, 1.20)	-	-	-	-
Least-vulnerable counties	0.49 (0.47, 0.51)	Ref	-	-	-	-

MHSVI= Minority Health Social Vulnerability Index, PM= predicted marginals, aOR= adjusted odds ratio, CI= confidence interval, NH= Non-Hispanic, AA= African American, BMI = body mass index, Ref= reference group.
Most-vulnerable counties fall in the top quartile of the MHSVI, whereas least-vulnerable counties fall in the bottom quartile of MHSVI.
Bolded cells represent significant results.
Logistic regression analysis modeled the probability of 1= 1+ times of telehealth use.
Income and education were entered as ordinal variables; age and digital literacy were entered as continuous variables; all other variables (e.g., race/ethnicity, sex) were entered as categorical variables. Healthy BMI ranged from 18.5 to <25 kg/m^2, whereas unhealthy BMI was either <18.5 or ≥25 kg/m^2; sufficient physical activity was ≥150 minutes per week, whereas insufficient physical activity was <150 minutes per week; cigarette smoking, e-cigarette use, and marijuana use were coded "yes" when participants self-reported use on ≥1 days in the past month; alcohol misuse was defined as ≥3 drinks/day for men and ≥2 drinks/day for women, otherwise alcohol misuse was coded no.
Predicted marginals for digital health literacy domains recorded for 25th and 75th percentile of the score and for top age of all 4 age brackets.

[a] Non-Hispanic Asian, Native Hawaiian, Pacific Islander, American Indian, and Alaska Native individuals were collapsed into NH Other.

© 2025 Wakeman M et al. *JAMA Network Open.*

eTable 5. Associations between sociodemographic, health, and technology factors and telemedicine use (*n*= 4318, complete case).

	Overall (*N*= 4318)		MHSVI most-vulnerable counties (*n*= 2199)		MHSVI least-vulnerable counties (*n*= 2119)	
	PM (95% CI)	aOR (95% CI)	PM (95% CI)	aOR (95% CI)	PM (95% CI)	aOR (95% CI)
**Age**						
18	0.37 (0.34, 0.41)	**0.97 (0.97, 0.98)**	0.41 (0.36, 0.46)	**0.97 (0.96, 0.98)**	0.33 (0.28, 0.38)	**0.98 (0.97, 0.98)**
30	0.32 (0.30, 0.35)		0.36 (0.33, 0.39)		0.29 (0.26, 0.32)	
45	0.27 (0.25, 0.28)		0.30 (0.28, 0.32)		0.24 (0.22, 0.26)	
60	0.22 (0.20, 0.24)		0.24 (0.21, 0.27)		0.20 (0.17, 0.22)	
**Sex**						
Female	0.26 (0.25, 0.28)	0.94 (0.81, 1.11)	0.30 (0.27, 0.32)	0.94 (0.75, 1.17)	0.23 (0.21, 0.26)	0.94 (0.74, 1.19)
Male	0.27 (0.25, 0.29)	Ref	0.31 (0.28, 0.34)	Ref	0.24 (0.21, 0.27)	Ref
**Sexual orientation**						
Heterosexual	0.27 (0.25, 0.28)	Ref	0.30 (0.28, 0.32)	Ref	0.23 (0.21, 0.25)	Ref
Non-heterosexual	0.29 (0.25, 0.33)	1.13 (0.88, 1.43)	0.29 (0.24, 0.34)	0.93 (0.66, 1.30)	0.29 (0.23, 0.35)	1.43 (1.00, 2.03)
**Race and ethnicity**						
NH Black or AA	0.30 (0.27, 0.34)	**1.34 (1.07, 1.69)**	0.33 (0.29, 0.37)	1.32 (1.00, 1.74)	0.28 (0.21, 0.36)	1.42 (0.90, 2.23)
Hispanic or Latino	0.29 (0.26, 0.33)	**1.28 (1.02, 1.60)**	0.32 (0.28, 0.36)	1.27 (0.96, 1.68)	0.27 (0.21, 0.34)	1.27 (0.84, 1.92)
NH Other[a]	0.28 (0.22, 0.33)	1.14 (0.80, 1.62)	0.30 (0.23, 0.39)	1.13 (0.68, 1.88)	0.25 (0.18, 0.33)	1.15 (0.69, 1.89)
NH White	0.25 (0.24, 0.27)	Ref	0.28 (0.25, 0.31)	Ref	0.23 (0.21, 0.25)	Ref
**Education**						
<High school	0.23 (0.20, 0.26)	**1.15 (1.03, 1.27)**	0.25 (0.21, 0.29)	**1.19 (1.04, 1.36)**	0.20 (0.16, 0.26)	1.10 (0.94, 1.29)
High school graduate	0.25 (0.23, 0.27)		0.28 (0.25, 0.30)		0.22 (0.19, 0.25)	

© 2025 Wakeman M et al. *JAMA Network Open.*

	Overall (*N*= 4318)		MHSVI most-vulnerable counties (*n*= 2199)		MHSVI least-vulnerable counties (*n*= 2119)	
	PM (95% CI)	aOR (95% CI)	PM (95% CI)	aOR (95% CI)	PM (95% CI)	aOR (95% CI)
Vocational school, some college	0.27 (0.26, 0.28)		0.31 (0.29, 0.33)		0.23 (0.21, 0.25)	
College graduate or higher	0.29 (0.27, 0.31)		0.34 (0.30, 0.37)		0.25 (0.22, 0.28)	
**Income (in 2021)**						
<$20,000	0.28 (0.25, 0.30)	0.96 (0.89, 1.04)	0.30 (0.27, 0.33)	0.99 (0.88, 1.10)	0.25 (0.21, 0.29)	0.94 (0.83, 1.05)
$20,000 to $49,999	0.27 (0.26, 0.29)		0.30 (0.28, 0.32)		0.24 (0.22, 0.27)	
$50,000 to $74,999	0.26 (0.25, 0.28)		0.30 (0.28, 0.32)		0.23 (0.21, 0.25)	
≥$75,000	0.26 (0.24, 0.28)		0.30 (0.26, 0.34)		0.22 (0.20, 0.25)	
**English proficiency**						
Very well	0.27 (0.25, 0.28)	Ref	0.30 (0.28, 0.32)	Ref	0.23 (0.22, 0.25)	Ref
Well, not well, not at all	0.31 (0.26, 0.36)	1.27 (0.96, 1.69)	0.36 (0.30, 0.43)	1.46 (1.00, 2.14)	0.25 (0.18, 0.32)	1.08 (0.69, 1.69)
**Health insurance**						
Insured	0.27 (0.26, 0.28)	Ref	0.30 (0.28, 0.32)	Ref	0.23 (0.22, 0.25)	Ref
Uninsured	0.24 (0.21, 0.29)	0.84 (0.64, 1.12)	0.28 (0.23, 0.33)	0.86 (0.61, 1.20)	0.22 (0.16, 0.31)	0.93 (0.55, 1.57)
**General health**						
Excellent, very good, good	0.27 (0.25, 0.28)	Ref	0.30 (0.27, 0.32)	Ref	0.23 (0.21, 0.26)	Ref
Fair, poor	0.27 (0.24, 0.30)	1.02 (0.83, 1.26)	0.31 (0.27, 0.35)	1.08 (0.82, 1.44)	0.23 (0.19, 0.27)	0.98 (0.72, 1.34)
**Mental health**						
Excellent, very good, good	0.26 (0.24, 0.28)	Ref	0.30 (0.28, 0.32)	Ref	0.22 (0.20, 0.24)	Ref
Fair, poor	0.29 (0.26, 0.32)	1.18 (0.97, 1.44)	0.30 (0.27, 0.34)	1.04 (0.80, 1.35)	0.27 (0.23, 0.31)	**1.37 (1.02, 1.83)**
**Having a primary care clincian**						

© 2025 Wakeman M et al. *JAMA Network Open.*

	Overall (*N*= 4318)		MHSVI most-vulnerable counties (*n*= 2199)		MHSVI least-vulnerable counties (*n*= 2119)	
	**PM (95% CI)**	**aOR (95% CI)**	**PM (95% CI)**	**aOR (95% CI)**	**PM (95% CI)**	**aOR (95% CI)**
Yes	0.30 (0.28, 0.32)	Ref	0.34 (0.31, 0.37)	Ref	0.26 (0.23, 0.29)	Ref
No	0.23 (0.22, 0.25)	**0.65 (0.55, 0.77)**	0.26 (0.24, 0.29)	**0.61 (0.48, 0.77)**	0.20 (0.18, 0.23)	**0.69 (0.54, 0.88)**
**Presence of underlying medical conditions**						
Yes	0.27 (0.25, 0.29)	0.99 (0.83, 1.19)	0.30 (0.26, 0.33)	0.96 (0.74, 1.23)	0.24 (0.21, 0.27)	1.02 (0.79, 1.31)
No	0.27 (0.25, 0.29)	Ref	0.30 (0.28, 0.33)	Ref	0.23 (0.21, 0.26)	Ref
**Past year in-person visit(s) with health care clinician**						
0 times	0.11 (0.09, 0.14)	Ref	0.10 (0.07, 0.14)	Ref	0.12 (0.09, 0.17)	Ref
1+ times	0.30 (0.29, 0.32)	**3.87 (2.95, 5.09)**	0.34 (0.32, 0.36)	**5.58 (3.74, 8.32)**	0.26 (0.24, 0.28)	**2.70 (1.84, 3.94)**
**Ever tested positive for COVID-19**						
Yes	0.26 (0.24, 0.28)	0.93 (0.78, 1.10)	0.30 (0.26, 0.33)	0.96 (0.76, 1.21)	0.22 (0.19, 0.26)	0.90 (0.70, 1.17)
No	0.27 (0.26, 0.29)	Ref	0.30 (0.28, 0.32)	Ref	0.24 (0.22, 0.26)	Ref
**BMI**						
Healthy	0.25 (0.23, 0.28)	Ref	0.28 (0.25, 0.31)	Ref	0.23 (0.20, 0.26)	Ref
Unhealthy	0.27 (0.26, 0.29)	1.14 (0.96, 1.34)	0.31 (0.29, 0.33)	1.20 (0.95, 1.51)	0.24 (0.21, 0.26)	1.04 (0.82, 1.33)
**Physical activity**						
Sufficient	0.28 (0.27, 0.30)	Ref	0.32 (0.30, 0.34)	Ref	0.24 (0.22, 0.27)	Ref
Insufficient	0.23 (0.21, 0.26)	**0.73 (0.61, 0.86)**	0.26 (0.22, 0.29)	**0.67 (0.53, 0.86)**	0.21 (0.18, 0.24)	0.80 (0.63, 1.03)
**Past month cigarette smoking**						
Yes	0.33 (0.30, 0.36)	**1.67 (1.38, 2.01)**	0.36 (0.32, 0.39)	**1.70 (1.32, 2.19)**	0.29 (0.25, 0.34)	**1.61 (1.21, 2.15)**

© 2025 Wakeman M et al. *JAMA Network Open.*

	Overall (*N*= 4318)		MHSVI most-vulnerable counties (*n*= 2199)		MHSVI least-vulnerable counties (*n*= 2119)	
	PM (95% CI)	aOR (95% CI)	PM (95% CI)	aOR (95% CI)	PM (95% CI)	aOR (95% CI)
No	0.24 (0.22, 0.26)	Ref	0.26 (0.24, 0.29)	Ref	0.22 (0.20, 0.24)	Ref
**Past month e-cigarette use**						
Yes	0.35 (0.32, 0.39)	**1.78 (1.44, 2.20)**	0.38 (0.33, 0.43)	**1.76 (1.32, 2.34)**	0.31 (0.26, 0.37)	**1.73 (1.24, 2.42)**
No	0.25 (0.24, 0.26)	Ref	0.28 (0.26, 0.30)	Ref	0.22 (0.20, 0.24)	Ref
**Alcohol misuse**						
Yes	0.26 (0.21, 0.31)	0.93 (0.68, 1.26)	0.30 (0.24, 0.37)	1.00 (0.66, 1.50)	0.21 (0.15, 0.28)	0.84 (0.52, 1.35)
No	0.27 (0.26, 0.28)	Ref	0.30 (0.28, 0.32)	Ref	0.24 (0.22, 0.26)	Ref
Not applicable (<21 years)	0.25 (0.19, 0.31)	0.86 (0.58, 1.29)	0.29 (0.20, 0.39)	0.92 (0.51, 1.65)	0.21 (0.14, 0.30)	0.82 (0.46, 1.47)
**Past month marijuana or cannabis use**						
Yes	0.34 (0.31, 0.37)	**1.71 (1.42, 2.06)**	0.38 (0.34, 0.42)	**1.90 (1.48, 2.46)**	0.29 (0.25, 0.33)	**1.52 (1.14, 2.01)**
No	0.24 (0.23, 0.26)	Ref	0.26 (0.24, 0.29)	Ref	0.22 (0.20, 0.24)	Ref
**Have internet access**						
Yes	0.27 (0.25, 0.28)	Ref	0.30 (0.28, 0.32)	Ref	0.23 (0.22, 0.25)	Ref
No	0.26 (0.17, 0.38)	0.94 (0.48, 1.84)	0.26 (0.16, 0.39)	0.76 (0.35, 1.65)	0.29 (0.13, 0.52)	1.41 (0.43, 4.58)
**Digital health literacy**						
Using technology to process health information	0.23 (0.21, 0.25)	**1.76 (1.32, 2.36)**	0.26 (0.23, 0.29)	**1.75 (1.17, 2.62)**	0.20 (0.17, 0.23)	**1.75 (1.13, 2.70)**
	0.28 (0.27, 0.30)		0.32 (0.29, 0.34)		0.25 (0.23, 0.28)	
Understanding of health concepts and language	0.27 (0.26, 0.29)	0.85 (0.65, 1.11)	0.31 (0.28, 0.33)	0.89 (0.61, 1.30)	0.24 (0.22, 0.27)	0.85 (0.57, 1.26)
	0.26 (0.25, 0.28)		0.30 (0.28, 0.32)		0.23 (0.21, 0.25)	
Ability to actively engage with digital services	0.29 (0.27, 0.31)	**0.72 (0.58, 0.91)**	0.33 (0.30, 0.36)	**0.71 (0.51, 0.98)**	0.26 (0.23, 0.29)	0.73 (0.52, 1.02)

© 2025 Wakeman M et al. *JAMA Network Open.*

	Overall (*N*= 4318)		MHSVI most-vulnerable counties (*n*= 2199)		MHSVI least-vulnerable counties (*n*= 2119)	
	PM (95% CI)	aOR (95% CI)	PM (95% CI)	aOR (95% CI)	PM (95% CI)	aOR (95% CI)
	0.26 (0.25, 0.28)		0.29 (0.27, 0.31)		0.23 (0.21, 0.25)	
Feel safe and in control	0.27 (0.25, 0.28)	1.03 (0.86, 1.23)	0.30 (0.27, 0.32)	1.06 (0.82, 1.38)	0.23 (0.21, 0.26)	0.98 (0.76, 1.27)
	0.27 (0.25, 0.28)		0.30 (0.28, 0.33)		0.23 (0.21, 0.26)	
Motivated to engage with digital services	0.24 (0.22, 0.27)	**1.45 (1.08, 1.94)**	0.28 (0.25, 0.31)	1.34 (0.89, 2.01)	0.21 (0.18, 0.24)	**1.58 (1.03, 2.41)**
	0.28 (0.26, 0.30)		0.31 (0.29, 0.33)		0.25 (0.22, 0.27)	
Access to digital services that work	0.28 (0.26, 0.30)	0.84 (0.63, 1.12)	0.31 (0.28, 0.35)	0.80 (0.53, 1.21)	0.24 (0.21, 0.27)	0.90 (0.60, 1.36)
	0.26 (0.25, 0.28)		0.30 (0.27, 0.32)		0.23 (0.21, 0.25)	
Digital services that suit individual needs	0.25 (0.23, 0.27)	1.23 (0.97, 1.57)	0.29 (0.26, 0.32)	1.12 (0.81, 1.54)	0.21 (0.19, 0.24)	**1.46 (1.01, 2.11)**
	0.27 (0.26, 0.28)		0.30 (0.28, 0.32)		0.24 (0.22, 0.26)	
**MHSVI**						
Most-vulnerable counties	0.27 (0.25, 0.29)	1.03 (0.87, 1.22)	-	-	-	-
Least-vulnerable counties	0.26 (0.25, 0.28)	Ref	-	-	-	-

MHSVI= Minority Health Social Vulnerability Index, PM= predicted marginals, aOR= adjusted odds ratio, CI= confidence interval, NH= Non-Hispanic, AA= African American, BMI = body mass index, Ref= reference group.
Most-vulnerable counties fall in the top quartile of the MHSVI, whereas least-vulnerable counties fall in the bottom quartile of MHSVI.
Bolded cells represent significant results.
Logistic regression analysis modeled the probability of 1= 1+ times of telemonitoring use.
Income and education were entered as ordinal variables; age and digital literacy were entered as continuous variables; all other variables (e.g., race/ethnicity, sex) were entered as categorical variables. Healthy BMI ranged from 18.5 to <25 kg/m^2, whereas unhealthy BMI was either <18.5 or ≥25 kg/m^2; sufficient physical activity was ≥150 minutes per week, whereas insufficient physical activity was <150 minutes per week; cigarette smoking, e-cigarette use, and marijuana use were coded "yes" when participants self-reported use on ≥1 days in the past month; alcohol misuse was defined as ≥3 drinks/day for men and ≥2 drinks/day for women, otherwise alcohol misuse was coded no.
Predicted marginals for digital health literacy domains recorded for 25th and 75th percentile of the score and for top age of all 4 age brackets.

[a] Non-Hispanic Asian, Native Hawaiian, Pacific Islander, American Indian, and Alaska Native individuals were collapsed into NH Other.

© 2025 Wakeman M et al. *JAMA Network Open.*

eTable 6. Associations between sociodemographic, health, and technology factors and telemonitoring use (*n*= 4318, complete case).

	Overall (*N*= 4318)		MHSVI most-vulnerable counties (*n*= 2199)		MHSVI least-vulnerable counties (*n*= 2119)	
	PM (95% CI)	aOR (95% CI)	PM (95% CI)	aOR (95% CI)	PM (95% CI)	aOR (95% CI)
**Age**						
18	0.18 (0.15, 0.21)	**0.98 (0.97, 0.99)**	0.24 (0.20, 0.29)	**0.98 (0.96, 0.99)**	0.11 (0.08, 0.15)	0.99 (0.98, 1.00)
30	0.16 (0.14, 0.18)		0.21 (0.18, 0.24)		0.11 (0.09, 0.13)	
45	0.14 (0.13, 0.15)		0.17 (0.15, 0.19)		0.10 (0.09, 0.11)	
60	0.11 (0.10, 0.13)		0.13 (0.11, 0.16)		0.09 (0.07, 0.11)	
**Sex**						
Female	0.13 (0.12, 0.15)	0.88 (0.72, 1.07)	0.18 (0.16, 0.20)	1.04 (0.80, 1.35)	0.08 (0.07, 0.10)	**0.68 (0.50, 0.93)**
Male	0.14 (0.13, 0.16)	Ref	0.17 (0.15, 0.20)	Ref	0.11 (0.10, 0.14)	Ref
**Sexual orientation**						
Heterosexual	0.14 (0.13, 0.15)	Ref	0.18 (0.16, 0.20)	Ref	0.09 (0.08, 0.11)	Ref
Non-heterosexual	0.14 (0.12, 0.18)	1.07 (0.79, 1.45)	0.16 (0.12, 0.20)	0.82 (0.55, 1.21)	0.14 (0.10, 0.19)	1.60 (1.00, 2.56)
**Race and ethnicity**						
NH Black or AA	0.16 (0.13, 0.19)	**1.37 (1.03, 1.81)**	0.19 (0.15, 0.22)	1.31 (0.95, 1.82)	0.13 (0.08, 0.20)	1.48 (0.79, 2.79)
Hispanic or Latino	0.17 (0.14, 0.20)	**1.49 (1.15, 1.94)**	0.21 (0.17, 0.24)	**1.54 (1.11, 2.12)**	0.12 (0.08, 0.18)	1.36 (0.82, 2.28)
NH Other[a]	0.14 (0.10, 0.19)	1.19 (0.79, 1.80)	0.19 (0.13, 0.26)	1.34 (0.78, 2.31)	0.09 (0.05, 0.16)	0.98 (0.49, 1.96)
NH White	0.12 (0.11, 0.14)	Ref	0.15 (0.13, 0.18)	Ref	0.09 (0.08, 0.11)	Ref
**Education**						
<High school	0.13 (0.11, 0.16)	1.03 (0.90, 1.17)	0.16 (0.12, 0.20)	1.08 (0.91, 1.28)	0.11 (0.07, 0.16)	0.94 (0.76, 1.16)
High school graduate	0.13 (0.12, 0.15)		0.17 (0.15, 0.19)		0.10 (0.08, 0.13)	

© 2025 Wakeman M et al. *JAMA Network Open.*

	Overall (*N*= 4318)		MHSVI most-vulnerable counties (*n*= 2199)		MHSVI least-vulnerable counties (*n*= 2119)	
	**PM (95% CI)**	**aOR (95% CI)**	**PM (95% CI)**	**aOR (95% CI)**	**PM (95% CI)**	**aOR (95% CI)**
Vocational school, some college	0.14 (0.13, 0.15)		0.18 (0.16, 0.19)		0.10 (0.09, 0.11)	
College graduate or higher	0.14 (0.12, 0.16)		0.19 (0.16, 0.22)		0.09 (0.08, 0.12)	
**Income (in 2021)**						
<$20,000	0.13 (0.11, 0.15)	1.05 (0.95, 1.16)	0.17 (0.14, 0.19)	1.05 (0.93, 1.20)	0.09 (0.07, 0.11)	1.08 (0.92, 1.26)
$20,000 to $49,999	0.14 (0.12, 0.15)		0.17 (0.16, 0.19)		0.09 (0.08, 0.11)	
$50,000 to $74,999	0.14 (0.13, 0.15)		0.18 (0.16, 0.20)		0.10 (0.09, 0.11)	
≥$75,000	0.15 (0.13, 0.17)		0.19 (0.16, 0.22)		0.11 (0.09, 0.13)	
**English proficiency**						
Very well	0.13 (0.12, 0.14)	Ref	0.17 (0.15, 0.19)	Ref	0.09 (0.08, 0.11)	Ref
Well, not well, not at all	0.21 (0.17, 0.26)	**1.95 (1.40, 2.71)**	0.24 (0.19, 0.30)	**1.69 (1.12, 2.54)**	0.20 (0.13, 0.28)	**2.67 (1.52, 4.67)**
**Health insurance**						
Insured	0.14 (0.13, 0.15)	Ref	0.18 (0.16, 0.20)	Ref	0.10 (0.09, 0.11)	Ref
Uninsured	0.11 (0.09, 0.15)	0.75 (0.52, 1.08)	0.13 (0.10, 0.18)	0.66 (0.44, 1.00)	0.13 (0.07, 0.21)	1.40 (0.70, 2.79)
**General health**						
Excellent, very good, good	0.13 (0.12, 0.14)	Ref	0.17 (0.15, 0.18)	Ref	0.09 (0.08, 0.11)	Ref
Fair, poor	0.17 (0.14, 0.19)	**1.41 (1.08, 1.83)**	0.21 (0.17, 0.25)	**1.43 (1.02, 2.00)**	0.12 (0.09, 0.16)	1.41 (0.92, 2.15)
**Mental health**						
Excellent, very good, good	0.15 (0.14, 0.16)	Ref	0.19 (0.17, 0.21)	Ref	0.11 (0.09, 0.12)	Ref
Fair, poor	0.11 (0.09, 0.13)	**0.65 (0.50, 0.84)**	0.14 (0.11, 0.17)	**0.61 (0.44, 0.84)**	0.08 (0.06, 0.11)	0.75 (0.49, 1.15)
**Having a primary care clinician**						

© 2025 Wakeman M et al. *JAMA Network Open.*

	Overall (*N*= 4318)		MHSVI most-vulnerable counties (*n*= 2199)		MHSVI least-vulnerable counties (*n*= 2119)	
	PM (95% CI)	aOR (95% CI)	PM (95% CI)	aOR (95% CI)	PM (95% CI)	aOR (95% CI)
Yes	0.15 (0.14, 0.17)	Ref	0.20 (0.18, 0.23)	Ref	0.11 (0.09, 0.13)	Ref
No	0.12 (0.11, 0.14)	**0.71 (0.58, 0.88)**	0.15 (0.13, 0.17)	**0.65 (0.50, 0.86)**	0.09 (0.07, 0.11)	0.79 (0.56, 1.11)
**Presence of underlying medical conditions**						
Yes	0.13 (0.12, 0.15)	0.93 (0.74, 1.17)	0.17 (0.14, 0.20)	0.93 (0.68, 1.25)	0.10 (0.08, 0.12)	0.93 (0.66, 1.32)
No	0.14 (0.13, 0.15)	Ref	0.18 (0.16, 0.20)	Ref	0.10 (0.09, 0.12)	Ref
**Past year in-person visit(s) with health care clinician**						
0 times	0.06 (0.04, 0.08)	Ref	0.07 (0.05, 0.11)	Ref	0.05 (0.03, 0.08)	Ref
1+ times	0.15 (0.14, 0.16)	**2.98 (2.09, 4.27)**	0.19 (0.18, 0.21)	**3.53 (2.19, 5.67)**	0.11 (0.09, 0.12)	**2.34 (1.36, 4.03)**
**Ever tested positive for COVID-19**						
Yes	0.16 (0.14, 0.18)	**1.28 (1.04, 1.58)**	0.19 (0.16, 0.22)	1.14 (0.86, 1.50)	0.13 (0.10, 0.15)	**1.56 (1.13, 2.15)**
No	0.13 (0.14, 0.18)	Ref	0.17 (0.15, 0.19)	Ref	0.09 (0.07, 0.10)	Ref
**BMI**						
Healthy	0.14 (0.12, 0.16)	Ref	0.18 (0.15, 0.21)	Ref	0.10 (0.08, 0.12)	Ref
Unhealthy	0.14 (0.13, 0.15)	0.99 (0.81, 1.21)	0.17 (0.15, 0.19)	0.93 (0.72, 1.21)	0.10 (0.08, 0.12)	1.01 (0.73, 1.39)
**Physical activity**						
Sufficient	0.15 (0.14, 0.17)	Ref	0.19 (0.17, 0.21)	Ref	0.11 (0.10, 0.13)	Ref
Insufficient	0.10 (0.08, 0.12)	**0.56 (0.44, 0.70)**	0.13 (0.10, 0.16)	**0.56 (0.41, 0.76)**	0.07 (0.05, 0.09)	**0.57 (0.39, 0.83)**
**Past month cigarette smoking**						
Yes	0.16 (0.15, 0.19)	**1.48 (1.17, 1.87)**	0.20 (0.17, 0.23)	**1.37 (1.02, 1.84)**	0.13 (0.10, 0.17)	**1.67 (1.13, 2.46)**

© 2025 Wakeman M et al. *JAMA Network Open.*

	Overall (*N*= 4318)		MHSVI most-vulnerable counties (*n*= 2199)		MHSVI least-vulnerable counties (*n*= 2119)	
	PM (95% CI)	aOR (95% CI)	PM (95% CI)	aOR (95% CI)	PM (95% CI)	aOR (95% CI)
No	0.12 (0.11, 0.14)	Ref	0.16 (0.14, 0.18)	Ref	0.09 (0.07, 0.10)	Ref
**Past month e-cigarette use**						
Yes	0.20 (0.17, 0.23)	**1.93 (1.51, 2.47)**	0.24 (0.20, 0.28)	**1.87 (1.38, 2.55)**	0.15 (0.11, 0.20)	**1.92 (1.27, 2.91)**
No	0.12 (0.11, 0.13)	Ref	0.15 (0.14, 0.17)	Ref	0.09 (0.07, 0.10)	Ref
**Alcohol misuse**						
Yes	0.14 (0.11, 0.18)	1.06 (0.73, 1.52)	0.17 (0.12, 0.23)	0.94 (0.58, 1.52)	0.11 (0.07, 0.17)	1.20 (0.69, 2.06)
No	0.14 (0.13, 0.15)	Ref	0.18 (0.16, 0.19)	Ref	0.10 (0.09, 0.11)	Ref
Not applicable (<21 years)	0.14 (0.10, 0.19)	0.99 (0.61, 1.61)	0.18 (0.12, 0.27)	1.04 (0.56, 1.91)	0.09 (0.04, 0.17)	0.91 (0.39, 2.09)
**Past month marijuana or cannabis use**						
Yes	0.17 (0.15, 0.19)	**1.47 (1.18, 1.84)**	0.21 (0.18, 0.25)	**1.57 (1.18, 2.09)**	0.12 (0.09, 0.15)	1.36 (0.94, 1.97)
No	0.12 (0.11, 0.14)	Ref	0.16 (0.14, 0.18)	Ref	0.09 (0.08, 0.11)	Ref
**Have internet access**						
Yes	0.14 (0.13, 0.15)	Ref	0.17 (0.16, 0.19)	Ref	0.10 (0.09, 0.11)	Ref
No	0.19 (0.11, 0.30)	1.56 (0.74, 3.30)	0.22 (0.12, 0.36)	1.40 (0.60, 3.28)	0.15 (0.05, 0.38)	1.78 (0.42, 7.39)
**Digital health literacy**						
Using technology to process health information	0.12 (0.10, 0.14)<br>0.14 (0.13, 0.15)	1.42 (0.98, 2.07)	0.16 (0.13, 0.19)<br>0.18 (0.16, 0.20)	1.29 (0.81, 2.06)	0.08 (0.06, 0.11)<br>0.10 (0.09, 0.12)	1.57 (0.83, 2.94)
Understanding of health concepts and language	0.14 (0.13, 0.16)<br>0.14 (0.13, 0.15)	0.82 (0.58, 1.16)	0.19 (0.16, 0.21)<br>0.17 (0.16, 0.19)	0.78 (0.51, 1.19)	0.10 (0.08, 0.12)<br>0.10 (0.09, 0.11)	0.94 (0.51, 1.72)
Ability to actively engage with digital services	0.17 (0.15, 0.19)	**0.58 (0.43, 0.77)**	0.21 (0.18, 0.24)	**0.58 (0.40, 0.83)**	0.13 (0.10, 0.16)	**0.54 (0.33, 0.89)**

© 2025 Wakeman M et al. *JAMA Network Open.*

	Overall (*N*= 4318)		MHSVI most-vulnerable counties (*n*= 2199)		MHSVI least-vulnerable counties (*n*= 2119)	
	PM (95% CI)	aOR (95% CI)	PM (95% CI)	aOR (95% CI)	PM (95% CI)	aOR (95% CI)
	0.13 (0.12, 0.14)		0.17 (0.16, 0.19)		0.10 (0.08, 0.11)	
Feel safe and in control	0.13 (0.11, 0.14)	1.27 (1.00, 1.61)	0.16 (0.14, 0.18)	**1.46 (1.05, 2.02)**	0.10 (0.08, 0.11)	1.08 (0.77, 1.52)
	0.14 (0.13, 0.15)		0.18 (0.17, 0.20)		0.10 (0.09, 0.12)	
Motivated to engage with digital services	0.12 (0.10, 0.14)	**1.49 (1.02, 2.19)**	0.15 (0.13, 0.19)	1.46 (0.90, 2.36)	0.08 (0.06, 0.11)	1.48 (0.79, 2.77)
	0.14 (0.13, 0.16)		0.18 (0.16, 0.20)		0.10 (0.09, 0.12)	
Access to digital services that work	0.13 (0.11, 0.15)	1.18 (0.80, 1.74)	0.17 (0.14, 0.20)	1.17 (0.70, 1.97)	0.09 (0.07, 0.12)	1.19 (0.65, 2.18)
	0.14 (0.13, 0.15)		0.18 (0.16, 0.19)		0.10 (0.09, 0.11)	
Digital services that suit individual needs	0.12 (0.10, 0.14)	**1.40 (1.04, 1.89)**	0.16 (0.13, 0.19)	1.30 (0.90, 1.89)	0.08 (0.06, 0.10)	**1.78 (1.04, 3.03)**
	0.14 (0.13, 0.15)		0.17 (0.16, 0.19)		0.10 (0.09, 0.12)	
**MHSVI**						
Most-vulnerable counties	0.15 (0.14, 0.17)	**1.42 (1.15, 1.75)**	-	-	-	-
Least-vulnerable counties	0.12 (0.10, 0.13)	Ref	-	-	-	-

MHSVI= Minority Health Social Vulnerability Index, PM= predicted marginals, aOR= adjusted odds ratio, CI= confidence interval, NH= Non-Hispanic, AA= African American, BMI = body mass index, Ref= reference group.
Most-vulnerable counties fall in the top quartile of the MHSVI, whereas least-vulnerable counties fall in the bottom quartile of MHSVI.
Bolded cells represent significant results.
Logistic regression analysis modeled the probability of 1= telemonitoring use.
Income and education were entered as ordinal variables; age and digital literacy were entered as continuous variables; all other variables (e.g., race/ethnicity, sex) were entered as categorical variables. Healthy BMI ranged from 18.5 to <25 kg/m^2, whereas unhealthy BMI was either <18.5 or ≥25 kg/m^2; sufficient physical activity was ≥150 minutes per week, whereas insufficient physical activity was <150 minutes per week; cigarette smoking, e-cigarette use, and marijuana use were coded “yes” when participants self-reported use on ≥1 days in the past month; alcohol misuse was defined as ≥3 drinks/day for men and ≥2 drinks/day for women, otherwise alcohol misuse was coded no.
Predicted marginals for digital health literacy domains recorded for 25th and 75th percentile of the score and for top age of all 4 age brackets.

[a] Non-Hispanic Asian, Native Hawaiian, Pacific Islander, American Indian, and Alaska Native individuals were collapsed into NH Other.

© 2025 Wakeman M et al. *JAMA Network Open.*
