# Supplementary material for: Disparities in Digital Health Care Use in 2022
Source: JAMA Netw Open. 2025 Apr 17;8(4):e255359. doi: 10.1001/jamanetworkopen.2025.5359 (PMC12550827; doi:10.1001/jamanetworkopen.2025.5359)
Supplement: Supplement 2. — Data Sharing Statement [file jamanetwopen-e255359-s002.pdf]

Data Sharing Statement

Wakeman. Disparities in Digital Health Care Use in 2022. *JAMA Netw Open*. Published April 16, 2025. doi:10.1001/jamanetworkopen.2025.5359

Data
Data available: Yes
Data types: Deidentified participant data
How to access data: Data are available upon request from the corresponding author (sherine.el-toukhy@nih.gov).
When available: With publication

Supporting Documents
Document types: None

Additional Information
Who can access the data: Researchers whose proposed use of the data has been approved.
Types of analyses: For a specified purpose.
Mechanisms of data availability: With a signed data access agreement.
